# Single-cell analyses of regulatory network perturbations using enhancer-targeting TALEs suggest novel roles for *PU.1* during haematopoietic specification

**DOI:** 10.1242/dev.115709

**Published:** 2014-10

**Authors:** Adam C. Wilkinson, Viviane K. S. Kawata, Judith Schütte, Xuefei Gao, Stella Antoniou, Claudia Baumann, Steven Woodhouse, Rebecca Hannah, Yosuke Tanaka, Gemma Swiers, Victoria Moignard, Jasmin Fisher, Shimauchi Hidetoshi, Marloes R. Tijssen, Marella F. T. R. de Bruijn, Pentao Liu, Berthold Göttgens

**Affiliations:** 1 Cambridge Institute for Medical Research and Wellcome Trust–MRC Cambridge Stem Cell Institute, University of Cambridge, Hills Road, Cambridge CB2 0XY, UK; 2 Division of Periodontology and Endodontology, Tohoku University Graduate School of Dentistry, Sendai 980-8575, Japan; 3 Wellcome Trust Sanger Institute, Cambridge CB10 1SA, UK; 4 MRC Molecular Haematology Unit, Weatherall Institute of Molecular Medicine, Radcliffe Department of Medicine, John Radcliffe Hospital, University of Oxford, Oxford OX3 9DS, UK; 5 Microsoft Research Cambridge, 21 Station Road, Cambridge CB1 2FB, UK; 6 Department of Biochemistry, University of Cambridge, Cambridge CB2 1QW, UK; 7 Department of Haematology, University of Cambridge and National Health Service Blood and Transplant, Cambridge CB2 0PT, UK

**Keywords:** Haematopoiesis, Transcription activator-like effectors, Regulatory networks, PU.1

## Abstract

Transcription factors (TFs) act within wider regulatory networks to control cell identity and fate. Numerous TFs, including Scl (Tal1) and PU.1 (Spi1), are known regulators of developmental and adult haematopoiesis, but how they act within wider TF networks is still poorly understood. Transcription activator-like effectors (TALEs) are a novel class of genetic tool based on the modular DNA-binding domains of *Xanthomonas* TAL proteins, which enable DNA sequence-specific targeting and the manipulation of endogenous gene expression. Here, we report TALEs engineered to target the *PU.1-14kb* and *Scl+40kb* transcriptional enhancers as efficient new tools to perturb the expression of these key haematopoietic TFs. We confirmed the efficiency of these TALEs at the single-cell level using high-throughput RT-qPCR, which also allowed us to assess the consequences of both *PU.1* activation and repression on wider TF networks during developmental haematopoiesis. Combined with comprehensive cellular assays, these experiments uncovered novel roles for *PU.1* during early haematopoietic specification. Finally, transgenic mouse studies confirmed that the *PU.1-14kb* element is active at sites of definitive haematopoiesis *in vivo* and PU.1 is detectable in haemogenic endothelium and early committing blood cells. We therefore establish TALEs as powerful new tools to study the functionality of transcriptional networks that control developmental processes such as early haematopoiesis.

## INTRODUCTION

Transcriptions factors (TFs) are key regulators of cell identity and fate. Cell type-specific transcriptional regulation is thought to largely occur by TF binding to distal *cis*-regulatory elements (Heinz et al., 2010). The haematopoietic system provides a well-studied model of mammalian tissue development, in which numerous key TFs have been described [reviewed by Wilkinson and Gottgens (2013)], including Scl (Tal1) and PU.1 (Spi1). The identification of *cis*-regulatory elements that regulate the expression of such TFs has begun to reveal TF circuits that suggest the existence of highly interconnected TF regulatory networks active in the haematopoietic system (Pimanda and Gottgens, 2010; Schutte et al., 2012).

Two well-studied examples of such haematopoietic *cis*-regulatory elements are the *PU.1-14kb* (Rosenbauer et al., 2004; Okuno et al., 2005; Huang et al., 2008; Staber et al., 2013) and *Scl+40kb* (Delabesse et al., 2005; Ogilvy et al., 2007; Ferreira et al., 2013). The *PU.1-14kb* plays a key role in *PU.1* expression in haematopoietic stem/progenitor cells (HSPCs) and mature haematopoietic cell types; its deletion results in an 80% loss of *PU.1* gene expression and acute myeloid leukaemia (AML) in mice (Rosenbauer et al., 2004), while mutation of an (autoregulatory) Ets site within the *PU.1-14kb* causes a 66% reduction in *PU.1* gene expression, which leads to haematopoietic stem cell exhaustion (Staber et al., 2013). Although the *Scl+40kb* element is active during haematopoietic emergence, its deletion causes only a mild erythroid phenotype (Ferreira et al., 2013). The *Scl+40kb* element is additionally thought to regulate expression of the 3′ flanking gene, *Map17* (*Pdzk1ip1*) (Tijssen et al., 2011; Ferreira et al., 2013).

Recent technological advances in microfluidic technology have led to the development of robust protocols for high-throughput quantification of gene expression in single cells (Guo et al., 2010). One of the earliest studies reporting microfluidics-based single-cell gene expression highlighted the potential for heterogeneity of knockdown efficiency within single cells following siRNA-mediated gene silencing (Toriello et al., 2008). However, the ability to accurately assess gene expression in single cells following conventional perturbations, such as retroviral overexpression or shRNA-mediated knockdown, has been limited because the former commonly yields unphysiologically high expression levels with no means to distinguish between the endogenous and ectopically expressed gene, whereas the latter acts post-transcriptionally and can therefore inhibit protein production without affecting transcript abundance. To realise the full potential of analysing perturbation phenotypes by single-cell gene expression profiling, more physiological means to tune gene expression levels are therefore required.

Transcription activator-like effectors (TALEs) are a novel class of TFs identified in the bacterial plant pathogen *Xanthomonas*, where they are secreted as virulence factors to modulate gene expression of the host plant (Boch and Bonas, 2010). TALEs have a unique modular DNA-binding domain consisting of 33-35 amino acid repeats, each of which binds a single nucleotide with base recognition specificity (Boch et al., 2009). TALEs fused to transcriptional effector domains have been shown to modulate endogenous gene expression (Zhang et al., 2011; Cong et al., 2012; Gao et al., 2013).

Here, we present the use of TALEs (fused to transcriptional effector domains) designed to target conserved regions within haematopoietic TF *cis*-regulatory elements as an efficient tool to regulate target gene expression. We validated TALEs targeting the *PU.1-14kb* and *Scl+40kb* elements and further assessed the phenotypic effect of modulating the activity of these enhancers on embryoid body (EB) haematopoiesis. We go on to highlight the combination of TALE-mediated endogenous gene expression perturbations with single-cell gene expression studies as a powerful approach to investigate TF regulatory networks. Using these methods in combination with transgenic embryo analysis, we uncover a novel role for PU.1 expression, mediated via *PU.1-14kb*, in haematopoietic specification during development.

## RESULTS

### Design and validation of TALEs targeting conserved regions within haematopoietic enhancers

**Fig. 1.**
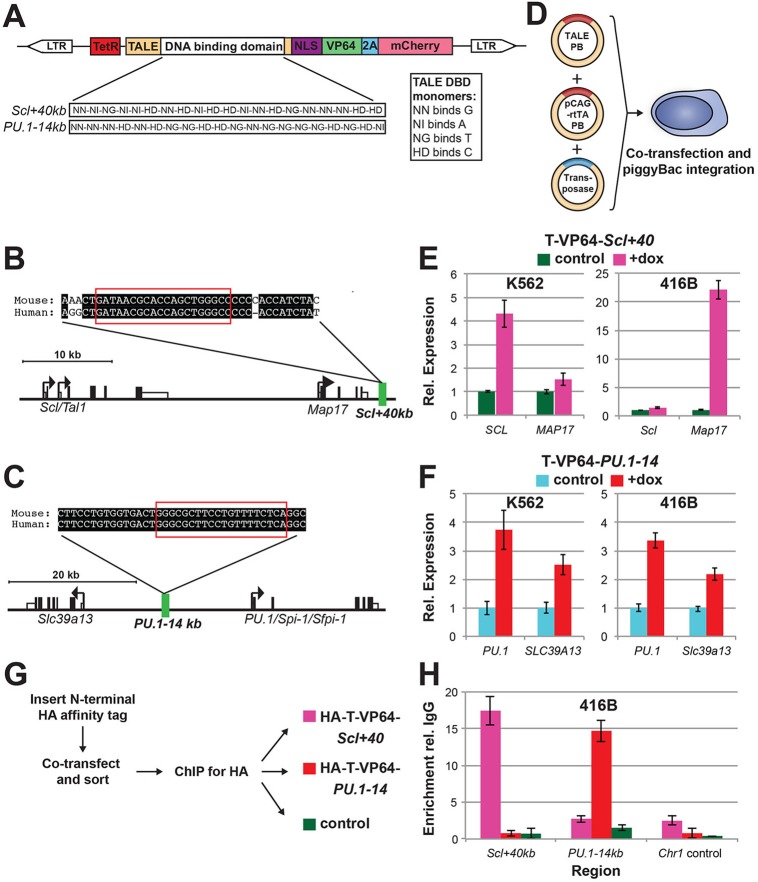
**Experimental approach and validation.** (A) Structure of the TALE-expressing piggyBac construct. TALE cDNA consists of the TALE sequence followed by a nuclear localisation domain (NLS), a VP64 domain, 2A (peptide sequence cleaved after translation) and mCherry fluorescent protein. TALE cDNA was cloned downstream of a tetracycline-responsive promoter (TetR), and within piggyBac long terminal repeats (LTRs) for stable transposase-mediated genomic integration. The DNA-binding domain (DBD) within the TALE sequence consists of twenty monomers. Monomers contain two hypervariable amino acids that determine nucleotide-binding specificity: NN, NI, NG or HD. (B,C) Schematics of the mouse *Scl* (*Tal1*) (B) and *PU.1* (*Spi1*) (C) genomic loci, with the *Scl+40kb* and *PU.1-14kb* elements highlighted in green. TALE target sites within conserved (between human and mouse) sequences are highlighted in red. (D) Experimental approach to express TALEs in cell lines. K562 and 416B cells were co-transfected with the TALE-expressing piggyBac (TALE-PB) from A, a constitutively expressing rtTA piggyBac vector (pCAG-rtTA-PB) and a piggyBac transposase, to create inducible TALE-expressing cells. (E) Effect of expressing TALE-VP64 targeting *Scl+40kb* (T-VP64-*Scl+40*) in human K562 (left) and mouse 416B (right) cells on neighbouring gene expression. T-VP64-*Scl+40* was expressed for 48 h by addition of doxycycline (dox) and gene expression in mCherry^+^ cells was determined relative to mCherry^−^ control cells. Error bars indicate s.d. of technical triplicates, and are representative of two biological replicates. (F) As in E, but for TALE-VP64 targeting *PU.1-14kb* (T-VP64-*PU.1-14*). (G) ChIP approach for TALE-VP64 proteins in H. An HA affinity tag was inserted at the N-terminus of the TALE-VP64 (HA-T-VP64); 416B cells were co-transfected as in D, sorted and ChIP performed 48 h after dox addition. (H) ChIP-qPCR enrichment of HA-tagged TALE-VP64 (HA-T-VP64) relative to IgG in HA-T-VP64-*Scl+40* (pink), HA-T-VP64-PU.1-14 (red) and untransfected 416B control (green) cells at *Scl+40kb*, *PU.1-14kb* and a control region on chromosome 1 (*Chr1*). Error bars indicate s.d. of technical triplicates from one biological experiment.

We identified regions within the *Scl+40kb* and *PU.1-14kb* elements that were perfectly conserved between human and mouse. TALEs were designed to match these regions and nowhere else in either genome (Fig. 1A-C). TALEs were initially assembled fused to the VP64 (transcriptional activator) domain (Beerli et al., 1998) and an mCherry fluorescent reporter via a 2A peptide (Fig. 1A). TALE constructs were cloned into piggyBac transposon-based plasmids (Wang et al., 2008) for efficient stable genomic integration and under the control of a tetracycline-responsive promoter (TetR) to provide inducible [with doxycycline (dox)] expression (Fig. 1A). We initially validated TALE-VP64 proteins in both human and mouse systems (Fig. 1D). In human K562 cells, the TALE-VP64 targeting *Scl+40kb* (T-VP64-*Scl+40*) upregulated *SCL* expression ∼4-fold but had little effect on *MAP17* expression (Fig. 1E). By contrast, in mouse 416B cells T-VP64-*Scl+40* upregulated *Map17* expression ∼22-fold but had little effect on *Scl* expression (Fig. 1E). In both the human K562 and mouse 416B cells, expression of the TALE-VP64 targeting *PU.1-14kb* (T-VP64-*PU.1-14*) upregulated *PU.1* expression 3- to 4-fold and *SLC39A13/Slc39a13* expression ∼2-fold (Fig. 1F).

Modest (1.5- to 8.5-fold) increases in histone H3 lysine 27 acetylation (H3K27Ac), an epigenetic modification associated with active regions of chromatin (Creyghton et al., 2010), were also seen in 416B cells at the promoters of TALE-VP64 target genes, consistent with increased transcription (supplementary material Fig. S1A,B). H3K27Ac was also enriched 3.8-fold at *Scl+40kb* when the TALE-VP64 targeting this enhancer was expressed (supplementary material Fig. S1A). However, a 50% reduction in H3K27Ac was seen at *PU.1-14kb* when the TALE-VP64 targeting this enhancer was expressed (supplementary material Fig. S1B), perhaps due to nucleosome displacement caused by TALE-VP64 and co-factor DNA binding. In mouse embryonic stem cells (mESCs), in which these enhancers are not active (as determined by H3K27Ac ChIP-seq enrichment; data not shown) and target genes are weakly expressed, TALE-VP64 did not upregulate gene expression (supplementary material Fig. S1C,D).

To determine the specificity of these TALEs, we further determined expression changes to genes within ∼100 kb of the target regions (supplementary material Fig. S1E-H). Less than 1.7-fold increases in expression were seen in K562, 416B and mESCs. Reduced expression in some genes (such as *Stil* in 416B cells expressing T-VP64-*Scl+40*) was identified, perhaps due to transcription factory reallocation (Papantonis and Cook, 2013). Additionally, we confirmed by chromatin immunoprecipitation (ChIP) that TALEs bind to their target regions. By ChIP-qPCR, enrichments of 15- to 17-fold were seen at target locations (Fig. 1G,H). To further assess TALE binding specificity genome-wide, we sequenced the HA-T-VP64-*PU.1-14* and 416B control HA antibody ChIP samples. The number of regions across the entire genome that showed enrichment was very small and comparable between the 416B control and HA-T-VP64-*PU.1-14* samples, underlining the high specificity afforded by TAL-mediated targeting reported by others (Mali et al., 2013). Importantly, a clear peak at the *PU.1-14kb* element could be identified in the HA-T-VP64-*PU.1-14* sample, but not in the control (supplementary material Fig. S1I). Manual assessment of enrichment at regions containing similar DNA sequences to the HA-T-VP64-*PU.1-14* target sequence did not identify strong off-target binding, and no other binding events occurred within a 15 Mb window around *PU.1-14kb* (supplementary material Fig. S1I).

**Fig. 2.**
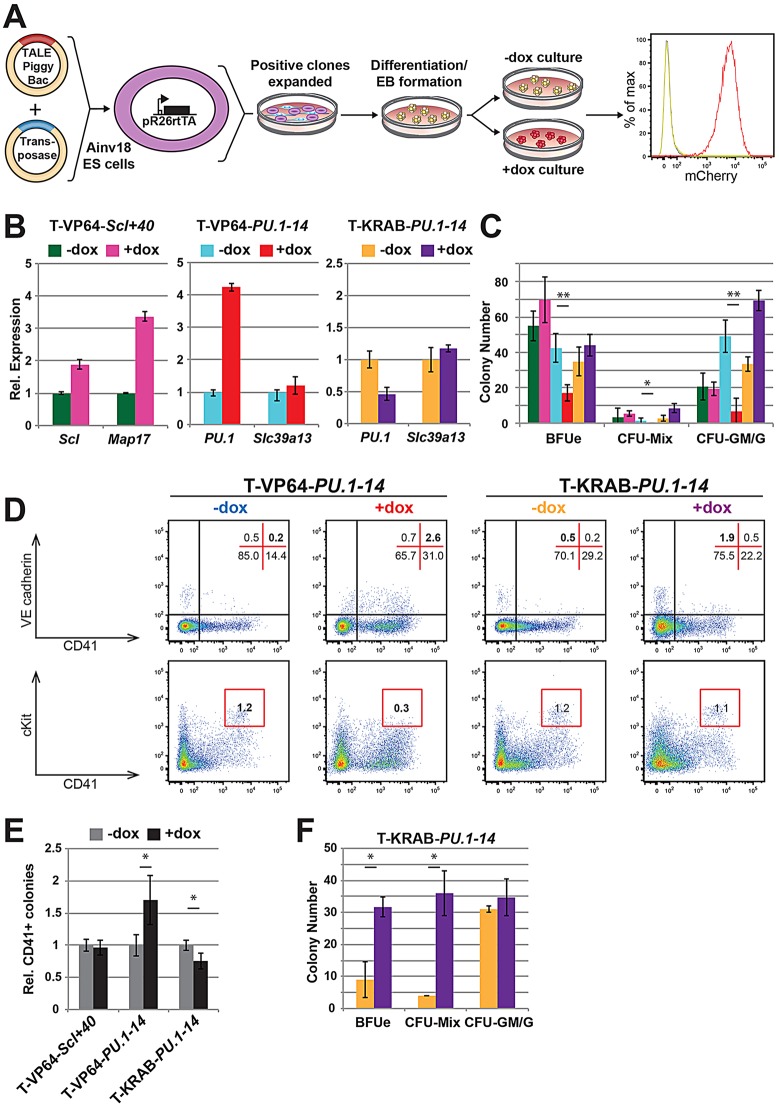
**Transient TALE expression affects haematopoietic cell fate decisions.** (A) Experimental approach using Ainv18 ESC differentiation to study TALE-mediated gene expression perturbations in haematopoiesis. Mouse Ainv18 ESCs constitutively expressing rtTA from the *Rosa26* locus (pR26-rtTA) were co-transfected with the inducible TALE-PB construct and transposase. Targeted ESCs were differentiated into embryoid bodies (EBs), and TALE expression induced at day 4 by addition of dox. Changes in gene expression, colony potential and surface marker phenotype were analysed at day 6 in the +dox EBs as compared with −dox controls. (B) Gene expression changes in day 6 EBs after induction of T-VP64-*Scl+40* (left), T-VP64-*PU.1-14* (middle) and T-KRAB-*PU.1-14* (right). Error bars indicate s.e.m. of three biological replicates. (C) Representative haematopoietic colony numbers from 1×10^5^ day 6 EB cells (colour scheme as in B). Colonies were grown in methylcellulose supplemented with SCF, IL-3, IL-6 and Epo. See supplementary material Fig. S2A for images of representative colony forming units (CFUs) scored. Error bars indicate s.d. of technical triplicates. *P<0.05, **P<0.01 (Student's *t*-test), from three biological replicates. (D) Flow cytometry plots of day 6 EB cells showing Flk1 versus CD41 (top) and VEcad versus CD41 (bottom). Representative staining patterns are shown for T-VP64-*PU.1-14* (left) and T-KRAB-*PU.1-14* (right). The distribution of cells within quadrants/gates is shown by percentage. (E) Relative number of day 4 Flk1**^+^** EB-derived colonies containing CD41^+^ haematopoietic cells, grown on OP9 stromal cells for 84 h (dox added after 36 h). See supplementary material Fig. S2G for representative image of scored colony. Error bars indicate s.e.m. from biological triplicates. *P<0.01 (Student's *t*-test), from three biological replicates. (F) Average numbers of haematopoietic colonies from 1×10^5^ day 6 EB T-KRAB*-PU.1-14* cells plated onto confluent OP9 stromal cells for 24 h before CFU assay initiated by addition of methylcellulose supplemented with SCF, IL-3, IL-6 and Epo. Colour scheme as in B. Error bars indicate s.d. of three biological replicates. *P<0.05 (Student's *t*-test), from three biological replicates.

We next assessed the ability of TALEs to regulate target gene expression during development using the mouse EB differentiation system, which has been validated as a useful and tractable *in vitro* model of embryonic haematopoiesis (Keller et al., 1993). We transfected the mESC line Ainv18 (Kyba et al., 2002), which constitutively expresses rtTA from the *Rosa26* locus, and expanded stably integrated clones that displayed inducible mCherry expression (data not shown). The data described below are representative of multiple clones tested for each TALE construct. We differentiated TALE-containing ESC lines, induced TALE expression by addition of dox at day 4 (just prior to definitive haematopoiesis in this system) and assessed phenotypic effects after a further 48 h of culture, relative to a culture without dox treatment (Fig. 2A). Initial flow cytometric analysis of the day 6 EBs confirmed pure mCherry^+^ populations in the dox cultures (Fig. 2A). Following this protocol, *Scl* expression was upregulated ∼1.9-fold in cells induced to express T-VP64-*Scl+40*, and *Map17* expression was upregulated over 3-fold (Fig. 2B). *PU.1* expression was upregulated over 4-fold by T-VP64-*PU.1-14*, with no significant change in *Slc39a13* expression (Fig. 2B). We additionally generated ESCs containing a *PU.1-14kb*-targeting TALE for *PU.1* repression by swapping the VP64 activation domain for the KRAB repressor domain (T-KRAB-*PU.1-14*). Following the same differentiation protocol as above, we observed efficient repression of *PU.1* by the TALE-KRAB, with expression reduced by over 50% (Fig. 2B). *Slc39a13* was unaffected by TALE expression, suggesting that, at least in this developmental context, *PU.1-14kb* activity is specific to *PU.1*.

### Transient expression of a *PU.1* enhancer-targeting TALE alters EB haematopoiesis

We next assessed the phenotypic effect of TALE-mediated modulation of gene expression by haematopoietic colony forming assays using day 6 EB cells (supplementary material Fig. S2A). TALE-VP64-mediated *PU.1* upregulation resulted in a significant loss of colony forming ability in day 6 EBs (Fig. 2C). By contrast, TALE-KRAB-mediated *PU.1* repression caused a doubling in myeloid (CFU-GM/G) and mixed (CFU-Mix) colony numbers (Fig. 2C), although this was not statistically significant. The colony potential of day 6 EBs was largely unaffected by expressing T-VP64-*Scl+40*, except for a slight (but not significant) increase in BFUe frequency (Fig. 2C).

To ensure that TALE-VP64 expression alone was not affecting CFU frequency, we assessed ESC lines carrying a non-functional TALE-VP64 [generated previously (Gao et al., 2013)], which did not affect CFU frequency (supplementary material Fig. S2B). To correlate the changes in haematopoietic progenitors/CFUs with changes in the cellular composition of the day 6 EBs, we analysed day 6 EBs by flow cytometry. Consistent with the modest effects in colony forming assays, expression of T-VP64-*Scl+40* minimally affected haematopoietic cell populations present in day 6 EBs (supplementary material Fig. S2C). Expression of TALEs targeting the *PU.1-14kb* partially, but not significantly, reduced the total cell numbers recovered (supplementary material Fig. S2D). However, this was not due to increased apoptosis, as assessed by Annexin V and DAPI staining of day 6 EBs (supplementary material Fig. S2D).

Although TALE-VP64-mediated upregulation of *PU.1* caused an increase in the relative size of the CD41^+^ population (Fig. 2D; supplementary material Fig. S2E), when combined with total cell numbers recovered from the EBs this did not result in a significant increase in the absolute number of CD41^+^ cells (supplementary material Fig. S2F). Interestingly, TALE-VP64-mediated *PU.1* expression caused a loss of the Flk1^+^ (mesoderm) population (supplementary material Fig. S2E,F), and significantly increased the CD41^+^ VE-cadherin (VEcad)^+^ (committing haemogenic endothelial) population (Fig. 2D; supplementary material Fig. S2F). Additionally, TALE-VP64-mediated *PU.1* upregulation caused a loss of the CD41^+^ cKit^hi^ (‘early definitive haematopoietic progenitor’) population, which might help explain the loss of colony forming potential described above (Fig. 2C,D). Combined with the CFU assays, these data suggested that PU.1 upregulation might push differentiating cells towards a haematopoietic fate but then inhibits proliferation of the resulting blood cells. Consistent with this hypothesis, TALE-mediated *PU.1* induction modestly increased (1.5-fold) day 4 EB-derived colonies containing budding CD41^+^ haematopoietic cells (Fig. 2E; supplementary material Fig. S2G), whereas *PU.1* repression modestly reduced their frequency.

By contrast, the major change caused by downregulation of *PU.1* by T-KRAB-*PU.1-14* was an almost complete loss of the CD45^+^ (Ptprc^+^) population (committed definitive haematopoietic cells; supplementary material Fig. S2E), which led us to speculate that the delayed haematopoiesis caused by *PU.1* repression might be masking an increase in haematopoietic CFU frequency. To test this further, we allowed day 6 EB cells to mature on OP9 stromal cells for 24 h before assessing CFU frequency (Fig. 2F). This led to a significant 3-fold and 9-fold increase in BFUe and CFU-Mix colonies, respectively, which is consistent with published data suggesting that PU.1 expression restricts haematopoietic cells to a myeloid fate (Mak et al., 2011).

Combined, these data suggest that upregulation of *PU.1* drives haematopoietic commitment, but causes loss of proliferative ability within the haematopoietic population, whereas temporary downregulation of *PU.1* inhibits the maturation and differentiation of early haematopoietic cells.

### Single-cell gene expression analysis of TALE-mediated *PU.1* perturbation

**Fig. 3.**
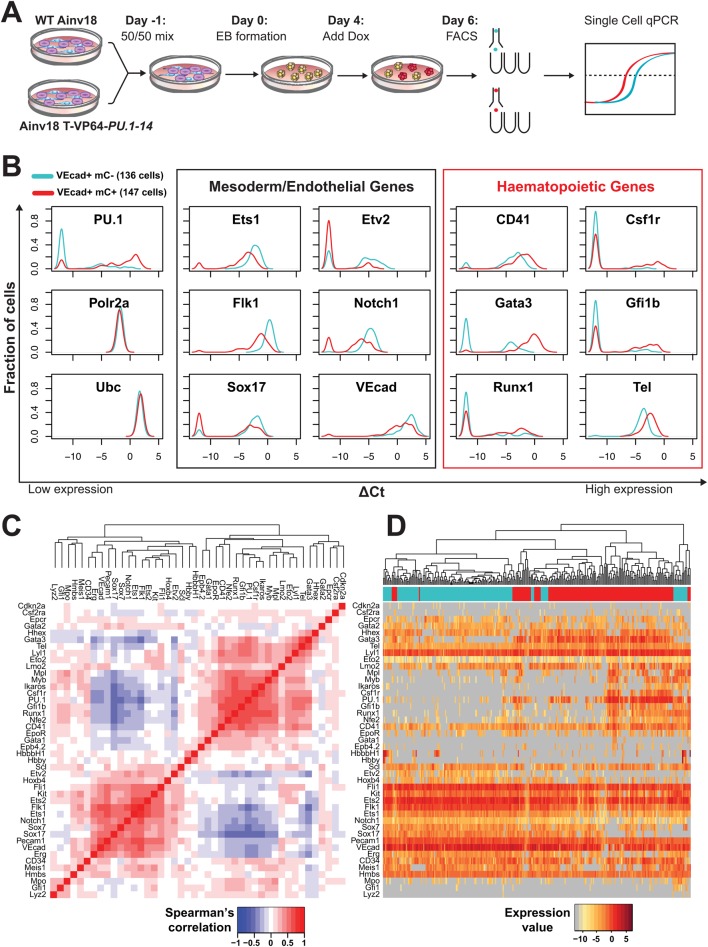
**Single-cell analysis of TALE-mediated *PU.1* expression in haematopoietic precursors.** (A) Strategy for single-cell gene expression analysis of TALE-mediated perturbations. Wild-type (WT) Ainv18 and T-VP64-*PU.1-14*-targeted ESCs were passaged once as a 1:1 mix before EB formation. Dox was added at day 4 and EBs disaggregated at day 6. Single VEcad^+^ cells (mCherry^+^ and mCherry^−^ sorted as T-VP64-*PU.1-14*-expressing and WT, respectively) were sorted into lysis buffer. Single-tube reverse transcription and targeted pre-amplification were undertaken, followed by multiplexed qPCR gene expression analysis using the Fluidigm Biomark platform. (B) Density plots of gene expression in day 6 EB VEcad^+^ mCherry^−^ (136 WT Ainv18; cyan) and VEcad^+^ mCherry^+^ (147 T-VP64-*PU.1-14* expressing; red) cells. The density indicates the fraction of cells at each expression level, relative to housekeeping genes (*Polr2a* and *Ubc*). Cells with non-detected gene expression were set to –12. See supplementary material Fig. S3 for density plots for all 48 genes analysed in these two populations. (C) Hierarchical clustering of Spearman rank correlations between all pairs of genes (excluding housekeepers) from all 283 VEcad^+^ cells (red, positive correlation; blue, negative correlation). (D) Hierarchical clustering of the 283 VEcad^+^ cells according to gene expression, with genes ordered according to C (dark red, highly expressed; grey, non-expressed). Top bar indicates cell type: cyan, mCherry^−^; red, mCherry^+^.

Having determined the phenotypic effects of TALE-mediated *PU.1* expression perturbations, we next asked what effects *PU.1* modulation might have on TF regulatory networks. We assessed the effect of T-VP64-*PU.1-14* induction on the expression of 44 haematopoietic, mesodermal and endothelial TFs and surface markers as well as four control housekeeping genes in single day-6 EB VEcad^+^ cells using the Fluidigm Biomark platform. At this time point, VEcad expression marks endothelium and haemogenic endothelium, which were not expected to express robust levels of *PU.1*. To provide an internal control, we differentiated a chimeric mixture of wild-type (WT) and TALE-inducible ESCs, and sorted VEcad^+^ cells from mCherry^−^ and mCherry^+^ populations at day 6 (48 h after dox addition; Fig. 3A). We assessed the expression of all 48 genes in 160 single cells for each population, which, after quality control, resulted in expression data for 136 and 147 cells, respectively.

*PU.1* was only expressed in 33% (45 of 136) of mCherry^−^ VEcad^+^ cells (Fig. 3B). By contrast, TALE-VP64 efficiently induced *PU.1* expression in 84% (124 of 147) of the mCherry^+^ VEcad^+^ cells. Moreover, *PU.1-*expressing cells in the mCherry^+^ VEcad^+^ population tended to express *PU.1* at a higher level than the *PU.1-*expressing cells in the mCherry^−^ VEcad^+^ population (an average of 3.3 ΔCt higher, relative to *Polr2a* and *Ubc* expression; Fig. 3B). This demonstrates that T-VP64-*PU.1-14* can induce gene expression efficiently (but not with complete efficiency), and that the distribution of *PU.1* expression levels within *PU.1*-expressing cells is altered, with a much larger proportion of individual cells expressing high levels of *PU.1*. Single-cell expression analysis therefore reveals both qualitative (a shift towards more cells expressing) and quantitative (a shift towards higher per-cell expression levels) consequences of TALE-mediated activation of *PU.1*.

Importantly, TALE-induced *PU.1* expression was associated with consistent changes in the expression of other genes. mCherry^+^ cells expressed higher levels of several haematopoietic genes, including *Csf1r*, *Gata3*, *Gfi1b*, *Runx1* and *Etv6* (*Tel*) (Fig. 3B). Interestingly, mCherry^+^ cells also express lower levels of several genes thought to be important for mesoderm or endothelium, including *Ets1*, *Etv2*, *Flk1* (*Kdr*), *Notch1*, *Sox17* and *VEcad* (*Cdh5*) (Fig. 3B). Moreover, gene expression changes for *Flk1*, *CD41* (*Itga2b*) and *Kit* correlated well with the expression of these surface markers as assessed by flow cytometry (Fig. 2D and Fig. 3B; supplementary material Figs S2 and S3). As *Kit* encodes the receptor for the pro-proliferative cytokine stem cell factor (Scf; Kitl), its downregulation at the transcriptional level and cell surface might partially explain the loss of proliferative ability in T-VP64-*PU.1-14*-expressing day 6 EB cells (Fig. 2E).

Pairwise all-against-all comparisons of the expression of the 44 TFs and surface proteins across all 283 single cells were performed by calculating Spearman rank correlation coefficients, which were displayed using a heatmap to illustrate both positive and negative correlations between pairs of genes. This identified two positively correlated gene clusters: a haematopoietic gene cluster (including *PU.1*) and a mesodermal/endothelial gene cluster (Fig. 3C). Although genes from both clusters can be co-expressed in single cells (Fig. 3D), genes from the haematopoietic cluster predominantly showed negative correlation to genes from the endothelial cluster (Fig. 3C), suggesting an antagonism between these regulatory networks. Pairwise analysis and hierarchical clustering of cells based on their gene expression signatures largely separated the mCherry^−^ and mCherry^+^ cells within the VEcad^+^ population (Fig. 3D). As expected, it was within the mCherry^+^ population that the positively correlated cluster of haematopoietic genes was more frequently activated.

### *PU.1* can promote haematopoietic commitment of haemogenic endothelial precursors

**Fig. 4.**
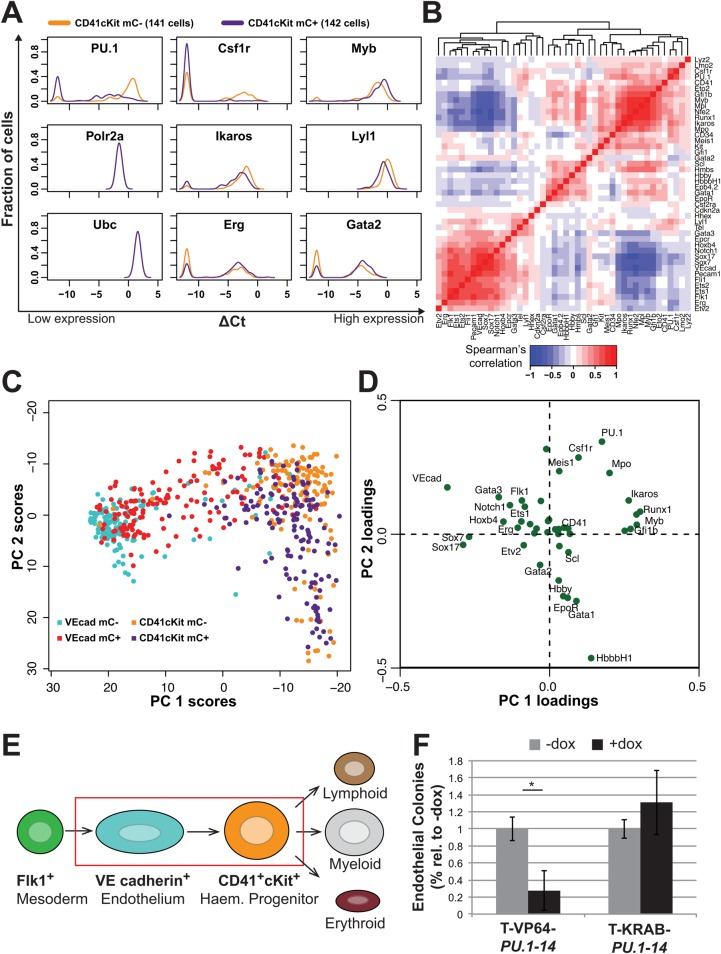
**TALE-mediated expression perturbations suggest transcriptional interactions during blood specification.** (A) Density plots of gene expression in day 6 EB CD41^+^ cKit^hi^ (CD41cKit) mCherry^−^ (141 WT Ainv18; orange) and CD41cKit mCherry^+^ (142 Ainv18 expressing T-KRAB-*PU.1-14*; purple) cells. The density indicates the fraction of cells at each expression level, relative to housekeeping genes (*Polr2a* and *Ubc*). Cells with non-detected gene expression were set to –12. See supplementary material Fig. S4 for density plots for all 48 genes analysed in these two populations. (B) Hierarchical clustering of Spearman rank correlations between all pairs of genes (excluding housekeepers) using gene expression data from all 566 cells (VEcad^+^ and CD41cKit). (C) Principal component analysis (PCA) of the 566 VEcad^+^ and CD41cKit cells, in the first and second components, from the expression of all 44 genes (excluding the four housekeeping genes). (D) Principal component loadings indicate the extent to which each gene contributes to the separation of cells along each component in C. (E) Current model of definitive haematopoietic specification from Flk1^+^ mesoderm through a haemogenic endothelial precursor to a haematopoietic stem/progenitor that can differentiate into lymphoid, myeloid or erythroid lineages. (F) Endothelial potential of TALE-expressing VEcad^+^ cells, as a percentage of –dox control cells. *P<0.01 (Student's *t*-test), from three biological replicates.

The data described above suggest that precocious *PU.1* expression in haematopoietic precursors can drive haematopoietic commitment through the activation of a TF network. To investigate this further, we performed additional single-cell gene expression analyses for the CD41^+^ cKit^hi^ population from WT and T-KRAB-*PU.1-14* differentiated ESCs. As above, 160 mCherry^+^ and mCherry^−^ cells were sorted from day 6 EBs, from which 142 and 141 single cells, respectively, passed quality control. Within the CD41^+^ cKit^hi^ mCherry^−^ (WT Ainv18) population, over 90% expressed *PU.1* (132 of 141), and clearly had acquired a committed haematopoietic gene expression pattern (including *Runx1*, *Myb*, *Ikaros*) with only a few cells expressing mesoderm/endothelium-associated genes (e.g. *Sox7*, *Sox17*, *Etv2*) (Fig. 4A; supplementary material Fig. S4). By contrast, less than 60% (85 of 142) of the CD41^+^ cKit^hi^ mCherry^+^ (TALE-KRAB-*PU.1-14*) cells expressed detectable *PU.1* transcript, and *PU.1* was expressed at lower levels in those that did (an average of 2.8 ΔCt lower; Fig. 4A), demonstrating that TALE-KRAB efficiently repressed *PU.1* expression in CD41^+^ cKit^hi^ cells. The expression of *Csf1r*, a known downstream target of PU.1, is tightly correlated with *PU.1* expression and *Csf1r* is not expressed in cells lacking *PU.1* (Fig. 4A). Other genes affected by repression of *PU.1* in CD41^+^ cKit^hi^ cells included the downregulation of *Ikaros* and *Lyl1*, as well as the upregulation of *Erg*, *Gata2* and *Myb* or an increase in the fraction of cells expressing the respective genes (Fig. 4A).

Having generated a total of 566 single-cell expression profiles from the TALE-VP64 and TALE-KRAB perturbation experiments, we next combined all the expression data to explore the potential of this substantial dataset for the identification of possible regulatory relationships. Pairwise all-against-all comparisons were performed as before by calculating Spearman rank correlation coefficients (Fig. 4B). This analysis placed *PU.1* next to a cluster of haematopoietic genes containing, among others, *Myb*, *Runx1* and *Ikaros*. A second cluster of strongly correlating genes consisted of endothelial genes (e.g. *Sox7*, *VEcad*, *Pecam1*). *Gata2* was adjacent to a third and somewhat smaller cluster consisting of erythroid genes such as *Gata1*, *Epb4.2* and globin genes. Of note, *PU.1* showed negative correlation with *Gata2*, as expected from the results in Fig. 4A, but not with core erythroid genes such as *Gata1*. Additionally, we observed negative correlation of *PU.1* with many genes within the ‘endothelial’ cluster, suggesting that *PU.1* might antagonise endothelial fate.

To further assess possible effects of *PU.1* expression perturbations on the entire multi-dimensional gene expression dataset from all 566 cells, we performed principal component analysis (PCA). PCA separated the two mCherry^−^ populations (VEcad^+^ and CD41^+^ cKit^hi^) into distinct groups across principal component 1 (PC1), consistent with the notion of two developmentally distinct populations (Fig. 4C). This separation is driven by the expression of endothelial genes in the VEcad^+^ population (including *VEcad*, *Sox17*, *Sox7*) and haematopoietic TFs in the CD41^+^ cKit^hi^ population (including *Runx1*, *Myb*, *Gfi1b*, *Ikaros* and *PU.1*) (Fig. 4D). The CD41^+^ cKit^hi^ population is resolved into two populations by PC2, by expression of myeloid genes (including *PU.1* and *Csf1r*) and erythroid genes (including *Hbb-bh1*, *Gata1*, *Epor*), suggesting that the CD41^+^ cKit^hi^ population contains myeloid- and erythroid-biased CD41^+^ cKit^hi^ progenitor cells. PCA of our dataset therefore provided good resolution of early developmental populations based on current models of developmental haematopoietic specification [Fig. 4E, based on Medvinsky et al. (2011)]. Interestingly, T-VP64-*PU.1-14* mCherry^+^ VEcad^+^ cells bridge the separation between the control VEcad^+^ and CD41^+^ cKit^hi^ populations (Fig. 4C), consistent with the notion that *PU.1* expression pushes VEcad^+^ cells to haematopoietic commitment but is unable to drive the transition completely. By contrast, the separation of the T-KRAB-*PU.1-14* mCherry^+^ CD41^+^ cKit^hi^ population from the mCherry^−^ CD41^+^ cKit^hi^ population is less striking, although more *PU.1* repressed cells are closer to the VEcad^+^ population and none form part of the most distant group of cells in the top right-hand part of the plot (Fig. 4C), consistent with the block observed in haematopoietic maturation.

Both the pairwise correlation analysis and PCA suggested that *PU.1* expression contributes to a haematopoietic fate in VEcad^+^ cells. We therefore assessed the effect of *PU.1* perturbation on the endothelial potential of the day 6 VEcad^+^ cells. TALE-VP64-mediated *PU.1* activation inhibited endothelial colony formation, whereas *PU.1* repression did not (Fig. 4F; supplementary material Fig. S5). Combined, these data suggest that activation of *PU.1* expression during developmental haematopoiesis plays a role in driving a haematopoietic rather than endothelial transcriptional programme, and that activation of *PU.1* expression in haemogenic endothelium might be an important molecular decision in haematopoietic commitment.

**Fig. 5.**
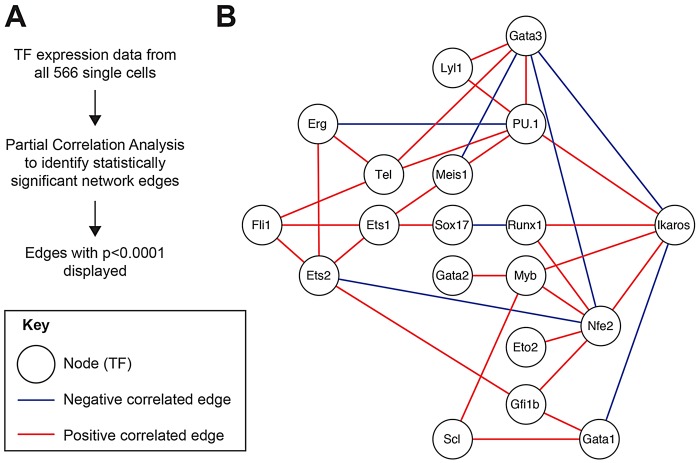
**Partial correlation analysis identifies a highly interconnected TF network that is active during the EHT.** (A) Method used to build the TF network model in B. (B) TF network model showing highly statistically significant interactions (*P*<0.0001) as connections (edges) between TFs (nodes).

Such a large single-cell gene expression dataset presented the opportunity to investigate underlying TF network interactions active during the endothelial-to-haematopoietic transition (EHT) using partial correlation analysis. This analysis identifies network interactions (edges) by detecting irreducible statistical dependencies between TFs that cannot be otherwise explained by other statistical dependencies within the network (see supplementary material Tables S1 and S2). To visualise the results, we plotted the 34 network edges between the TF nodes with highly significant correlations (*P*<0.0001; Fig. 5). Although this method of analysis provides positive/negative correlation information, directionality cannot be inferred. Most TF interactions were positive and formed a highly interconnected network, which could be important in network stabilisation. Two types of negative correlations were observed: (1) between haematopoietic genes and endothelial genes, including *Runx1* and *Sox17*; and (2) between haematopoietic lineage-specific genes, including *Nfe2* and *Gata3*. Such TF antagonisms might be important switches in cell fate commitment.

### The *PU.1-14kb* enhancer is active in the mid-gestation dorsal aorta *in vivo*

**Fig. 6.**
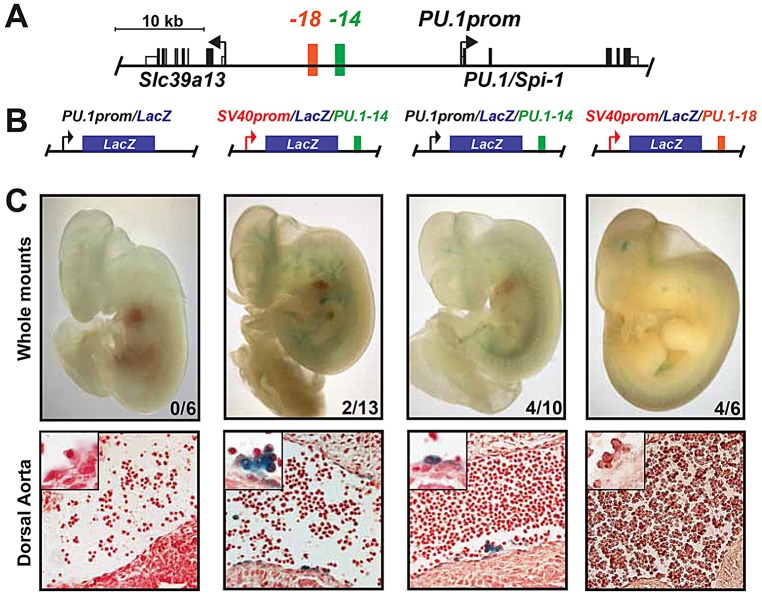
**The *PU.1-14kb* element is active at sites of mouse definitive haematopoiesis *in vivo.*** (A) Schematic of the *PU.1* locus highlighting the relevant *cis*-regulatory elements. (B) Reporter constructs used for transient transgenic embryo generation. (C) Representative *lacZ*^+^ whole-mount images and section images (original magnification: 40×) of the dorsal aorta of E11.5 transgenic embryos carrying the reporters illustrated above in B. Insets (original magnification: 100×) show cell clusters budding from the ventral side of the dorsal aorta. The number of *lacZ*^+^ embryos/number of total PCR^+^ embryos analysed is indicated in each whole-mount image.

The above data suggest that *PU.1* plays a role in driving endothelial cells to a haematopoietic fate. Although *PU.1* expression at sites of embryonic definitive haematopoiesis has been reported (Huang et al., 2008; Swiers et al., 2013), very little is known about its transcriptional regulation during developmental haematopoiesis *in vivo*. We therefore generated E11.5 transient transgenic mouse embryos carrying *lacZ* reporter gene constructs. The *PU.1* promoter alone (*PU.1prom/LacZ*) was unable to drive *lacZ* expression (Fig. 6A-C), suggesting that distal enhancers govern *PU.1* expression at this developmental stage. To determine the activity of the *PU.1-14kb* enhancer, we initially generated transgenic mouse embryos carrying the enhancer downstream of a *lacZ* reporter driven by the SV40 minimal promoter (*SV40prom/LacZ/PU.1-14*; Fig. 6B). In E11.5 mouse embryos, *PU.1-14kb* drove *lacZ* expression in cell clusters budding from the dorsal aorta (DA), a site of definitive haematopoiesis (Fig. 6C). *lacZ* expression was also seen in a minority of endothelial cells within the DA and rare circulating blood cells (Fig. 6C). These data demonstrate that *PU.1-14kb* is activated *in vivo* at embryonic sites of the EHT. By comparison, previous analysis of *Scl+40kb* failed to identify activity in the endothelium or budding clusters of the DA at E11.5, although primitive blood cells and HSPCs residing in the foetal liver later in development were *lacZ*^+^ (Ogilvy et al., 2007).

To confirm the ability of the *PU.1-14kb* enhancer to drive expression from the *PU.1* promoter, we also generated transient transgenic embryos carrying *PU.1-14kb* downstream of the *PU.1* promoter driving *lacZ* (*PU.1prom/LacZ/PU.1-14*; Fig. 6B). Embryos analysed at E11.5 also showed expression in cell clusters budding from the DA and endothelial patches of the DA, but expression in circulating cells was less obvious than with *SV40prom/LacZ/PU.1-14* (Fig. 6C). As a control, we generated transgenic embryos carrying a different putative *PU.1 cis*-regulatory element, *PU.1-18kb* (Chou et al., 2009; Zarnegar and Rothenberg, 2012), downstream of *lacZ* driven by the SV40 minimal promoter (*SV40/LacZ/PU.1-18*; Fig. 6A,B). E11.5 *SV40/LacZ/PU.1-18* embryos failed to display staining in the DA or circulating blood (Fig. 6C), although non-haematopoietic *lacZ* expression was detected (such as in the neural tube). We conclude that *PU.1-14kb* activates the *PU.1* promoter during the EHT, although other *cis*-regulatory elements are likely to be involved in regulating PU.1 expression during this process as *PU.1-14kb* is unable to confer physiological PU.1 expression in *PU.1*^−/−^ mice (Leddin et al., 2011).

### PU.1 is detectable within endothelium of the aorta-gonad-mesonephros region and vitelline and umbilical arteries, and is upregulated during the EHT *in vivo*

**Fig. 7.**
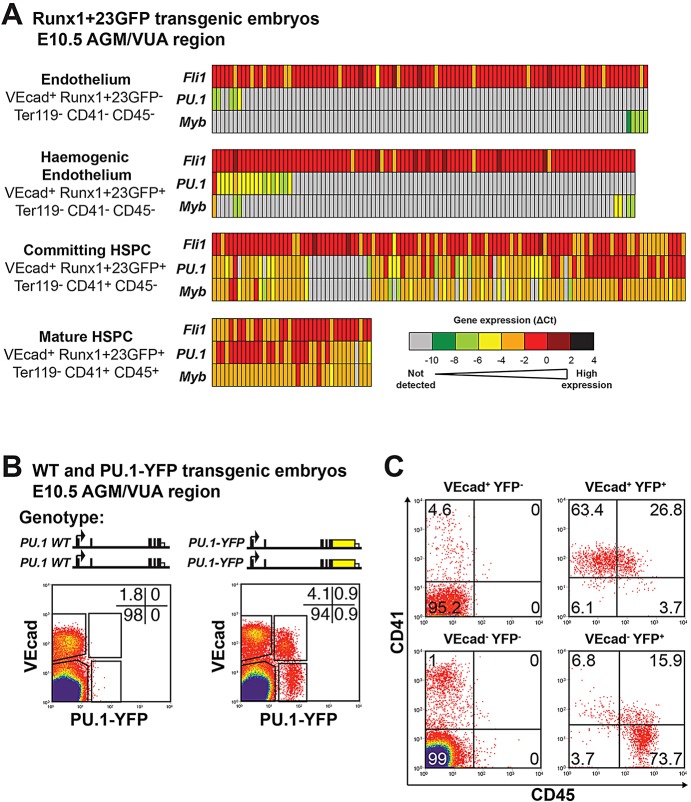
**PU.1 expression is induced during the EHT *in vivo.*** (A) *Fli1*, *PU.1* and *Myb* single-cell RT-qPCR gene expression data from Swiers et al. (2013) in the AGM region and vitelline and umbilical artery (AGM+VUA) for VEcad^+^ cell populations from E10.5 embryos carrying a Runx1+23kb-GFP enhancer reporter. Gene expression levels are displayed as a heatmap of ΔCt relative to housekeeping genes (*Ubc* and *Atp5a1*). (B) Genotype schematics and flow cytometry plots displaying VEcad versus PU.1-YFP expression for the Ter119^−^ (Ly76^−^) cell population from the AGM+VUA of E10.5 WT (left) and homozygous PU.1-YFP transgenic (right) embryos. Data were collected from pooled embryos and are representative of two E10-10.5 embryo litters. (C) Flow cytometry plots displaying CD41 versus CD45 expression for the populations within the VEcad/PU.1-YFP gates in B for the homozygous PU.1-YFP transgenic embryos.

Single-cell gene expression analysis has recently been used to study the EHT in the developing embryo (Swiers et al., 2013) using a *Runx1+23kb* enhancer-reporter (Runx1+23GFP) transgenic mouse line. Combined with surface markers, this reporter line allows further resolution of the cell types involved in the EHT: endothelium (VEcad^+^ Runx1+23GFP^−^ Ter119^−^ CD41^−^ CD45^−^), haemogenic endothelium (VEcad^+^ Runx1+23GFP^+^ Ter119^−^ CD41^−^ CD45^−^), committing HSPCs (VEcad^+^ Runx1+23GFP^+^ Ter119^−^ CD41^+^ CD45^−^) and mature HSPCs (VEcad^+^ Runx1+23GFP^+^ Ter119^−^ CD41^+^ CD45^+^). We re-analysed this dataset to specifically study *PU.1* expression during haematopoietic commitment in the aorta-gonad-mesonephros region and vitelline and umbilical arteries (AGM+VUA) from E10.5 embryos (Fig. 7A). As controls, we also re-analysed gene expression for *Fli1* (a TF expressed in endothelium and blood) and *Myb* (a haematopoietic TF). Low expression of *PU.1* was detectable in ∼5% of endothelium and ∼19% of haemogenic endothelium. Over 80% of early committing HSPCs expressed higher levels of *PU.1*, and by the mature HSPC stage 97% of cells expressed robust levels of *PU.1*. This expression profile was consistent at E8.5, E9.5 and E11.5 (data not shown) (Swiers et al., 2013). These expression dynamics were similar to those of the haematopoietic gene *Myb*, although it is interesting to note that, within the haemogenic endothelium population, only 7% of cells expressed *Myb*, which is lower than the 19% that were *PU.1* expressing. By the committing and mature HSPC states, *Myb* and *PU.1* expression almost entirely overlap. These data suggest that *in vivo*, *PU.1* can be expressed in the early haemogenic endothelial stages of the EHT, and is upregulated during this haematopoietic cell fate commitment, concomitant with CD41 surface expression.

Finally, to confirm this early expression of PU.1 at the protein level during the EHT, we analysed PU.1 expression in the AGM+VUA region of E10-10.5 *PU.1-YFP* knock-in homozygous mouse embryos (Kirstetter et al., 2006) by flow cytometry (Fig. 7B). PU.1-YFP was detectable in 0.6% of VEcad^+^ Ter119^−^ CD41^−^ CD45^−^ endothelium, 82% of VEcad^+^ Ter119^−^ CD41^+^ CD45^−^ committing HPSCs and 97% of VEcad^+^ Ter119^−^ CD41^+^ CD45^+^ mature HSPCs (Fig. 7B,C; supplementary material Fig. S6). These results correlate well with the *in vivo* and *in vitro* single-cell qPCR data (Fig. 3, Fig. 4 and Fig. 7A). Taken together, our results establish PU.1 and its *–14kb* enhancer as likely key building blocks of the gene regulatory network that drives early blood specification.

## DISCUSSION

Here, we demonstrate a novel use of TALEs in combination with single-cell gene expression profiling to investigate the consequences of transcriptional perturbation of developmental regulatory networks. High-throughput RT-qPCR coupled with comprehensive cellular assays uncovered a previously unrecognised role for *PU.1* in the EHT. Transgenic mouse studies confirmed that the *PU.1-14kb* enhancer is active at mid-gestation in the DA, where the EHT occurs *in vivo*, and that PU.1 expression can be detected from the endothelium stage of this process.

Our analysis of several hundred single cells with TALE induction highlights the efficiency of TALE-mediated endogenous gene expression perturbations at the single-cell level, which proved to be comparable to alternative methods of perturbation such as siRNA knockdown (Kouno et al., 2013), and provided more physiologically relevant expression changes when comparing upregulation of gene expression using TALE-VP64 proteins with retroviral cDNA overexpression. Moreover, TALE-mediated perturbation does not require a distinction between exogenous and endogenous cDNAs, allows normal co- and post-transcriptional processing to occur, and allows for detection by gene expression primers that are located in untranslated regions (UTRs).

The CRISPR-Cas9 system has recently been adapted to modulate gene expression by a similar mechanism to TALEs (Gilbert et al., 2013; Maeder et al., 2013; Perez-Pinera et al., 2013; Qi et al., 2013). As CRISPR-Cas9 target specificity is based on guide RNAs rather than a modular protein domain, the generation of these ‘designer’ TFs is faster than the assembly of TALEs (Gaj et al., 2013). However, a recent comparison between CRISPR-Cas9 and TALEs suggested higher targeting specificity for the latter (Fu et al., 2013; Mali et al., 2013).

The majority of previous research on PU.1 has concerned its role in adult haematopoiesis, where high PU.1 levels promote terminal myeloid differentiation, and reduced PU.1 expression results in proliferation [reviewed by Mak et al. (2011)], consistent with our CFU data. A novel link between PU.1 levels and the cell cycle has been described recently, in which PU.1, by regulating cell cycle lengthening, determines PU.1 protein accumulation within the cell, effecting lympho-myeloid cell fate decisions (Kueh et al., 2013). Our data additionally highlight the importance of tightly regulated *PU.1* expression for early haematopoiesis to occur. Moreover, since our TALEs target conserved DNA sequences within *cis*-regulatory elements, these tools can be directly applied to manipulate human haematopoiesis.

PU.1 has recently been shown to inhibit proliferation by directly controlling cell cycle regulators (Staber et al., 2013), which is consistent with our observed loss of haematopoietic colonies after TALE-VP64-mediated *PU.1* upregulation and increase in haematopoietic colonies after TALE-KRAB-mediated *PU.1* repression. Our gene selection for the single-cell expression analysis was focused on TF networks controlling early haematopoietic development, and it is likely that genes other than those assayed contribute to the phenotypic changes caused by *PU.1* expression perturbations, and this might well include cell cycle regulators. It is worth highlighting that the TALE-mediated perturbations caused consistent gene expression changes in single cells, suggesting that *PU.1* operates within a tightly interconnected haematopoietic TF network.

In adult haematopoiesis, *PU.1-14kb* is a known target of Runx1 (Huang et al., 2008), a TF crucial for definitive haematopoiesis (Okuda et al., 1996; Wang et al., 1996). We now report that the *PU.1-14kb* element is active *in vivo* in mid-gestation AGM endothelium and blood clusters, where definitive HSPCs arise, and PU.1 is expressed in a subset of these haematopoietic precursors. Runx1 has been shown to initiate chromatin unfolding at *PU.1-14kb* early during haematopoietic specification (Hoogenkamp et al., 2009). Such enhancer priming is likely to be important for efficient TALE-VP64-mediated induction of expression. Although Hoogenkamp et al. were unable to determine the frequency of such priming events within the precursor population, our data would be consistent with a model whereby the majority of VEcad^+^ cells contain primed *PU.1-14kb* enhancers owing to the high efficiency of TALE-mediated *PU.1* expression activation in this cell type. Enhancer priming might also contribute to the low-level expression of *PU.1* prior to haematopoietic commitment, with more robust expression seen later as haematopoietic TF network circuitry is reinforced. Such low-level expression might be analogous to the transcriptional noise of lineage regulators previously seen in adult haematopoietic progenitor cells (Pina et al., 2012).

In summary, we have validated the use of TALEs targeting conserved *cis*-regulatory elements as an efficient, multifaceted tool to modulate endogenous gene expression and study TF regulatory network perturbations in single cells, and in doing so we have uncovered a role for PU.1 in haematopoietic specification.

## MATERIALS AND METHODS

### TALE design and assembly

TALE sequences were designed to 20 bp regions within the *Scl+40kb* and *PU.1-14kb* elements that were conserved between human and mouse, and were unique within both genomes by BlastN and Blat (Altschul et al., 1990; Kent, 2002). TALEs were assembled and cloned into piggyBac (PB) as described previously (Gao et al., 2013). For ChIP experiments, an HA-tagged TALE-VP64 was used, as described previously (Gao et al., 2013).

### Cell culture and transfection

K562 (Lozzio et al., 1981) and 416B (Dexter et al., 1979) cells were cultured and electroporated as described previously (Knezevic et al., 2011; Moignard et al., 2013), as in Fig. 1D. TALE expression was induced by addition of 2 μg/ml dox (Sigma) for 48 h and mCherry^+^ and mCherry^–^ K562/416B cells were sorted using a BD Influx (BD Biosciences). Ainv18 ESCs (Kyba et al., 2002) were cultured as described previously (Ismailoglu et al., 2008) and transfected by nucleofection (Lonza). OP9 stromal cells were cultured as described previously (Nakano et al., 1994).

### ESC differentiation and haematopoietic colony forming assays

ESCs were differentiated essentially as described previously (Wilkinson et al., 2013), and treated as described in Fig. 2A and Fig. 3A. 100,000 day 6 EB cells were plated in triplicate in 1.1 ml M3434 Methocult (Stem Cell Technologies). For OP9 co-culture colony forming assays, 100,000 day 6 EB cells were plated on confluent OP9 in MEMα supplemented with 10% FCS for 24 h before the medium was replaced with M3434 Methocult. Definitive haematopoietic colonies were counted after 10-12 days (see supplementary material Fig. S2A for representative images of these colonies and counting criteria).

### Flow cytometry of EBs

Dissociated EB cells were stained with combinations of the antibodies listed in supplementary material Table S3. Annexin V-APC (BD Biosciences, 550474) antibody and DAPI were used to assess cell apoptosis according to the manufacturers' instructions. Surface marker and mCherry expression data were collected using a five-laser LSRFortessa (BD Biosciences) and analysed using FlowJo software.

### OP9 co-culture assays

MACS (Miltenyi) sorted Flk1^+^ cells from day 4 EBs (over 95% purity) were cultured on confluent OP9s in MEMα supplemented with 10% FCS. Mesodermal colonies were allowed to form for 36 h before dox was added. Haematopoietic cells were not seen before this time point. After 48 h, cells were fixed in 2% PFA overnight, blocked, stained with purified CD41 antibody (BD Biosciences, 553847, MWReg30), visualised by DAB staining, and mesodermal colonies containing (at least two) small rounded budding CD41^+^ haematopoietic cells were scored. Specific staining was confirmed using an isotype control antibody. VEcad^+^ cells from day 6 EBs were sorted using a BD Influx and plated on confluent OP9s, and cultured for 4 days in MEMα supplemented with 10% FCS. After 4 days, cells were fixed and stained as above using purified CD31 antibody (BD Biosciences, 553370, MEC13.3), and endothelial sheet colonies were scored.

### RNA extraction and RT-qPCR

RT-qPCR was undertaken as described previously (Wilson et al., 2010a) using the primers listed in supplementary material Table S4. All gene expression values are normalised to *ACTB*/*Actb*.

### Single-cell gene expression and data analysis

Single-cell sorting and gene expression analysis were undertaken as previously described (Moignard et al., 2013) using a BD Influx and TaqMan assays (see supplementary material Table S5). Partial correlation analysis, as described elsewhere (Bailey, 1995), was applied to the normalised TF gene expression data using Spearman correlation. Correlation values and associated *P*-values are displayed in supplementary material Tables S1 and S2, respectively. Interactions where *P*<0.0001 are displayed as edges in Fig. 6.

### ChIP

mCherry^+^ 416B cells were sorted by FACS (24 h after dox addition), then expanded without dox before HA-T-VP64 expression was induced for 48 h and cells crosslinked using 1% formaldehyde (Sigma). ChIP-qPCR and ChIP-seq were performed and analysed essentially as described previously (Wilson et al., 2010b) using anti-HA (Sigma, H6908) and anti-H3K27Ac (Abcam, ab4729) antibodies, and the ChIP-qPCR primers listed in supplementary material Table S6. ChIP-seq data are available at GEO with accession number GSE61189.

### Transient transgenic embryo generation

Promoter and enhancer reporter pGlac constructs were cloned as described previously (Wilson et al., 2009) using the primers listed in supplementary material Table S7. E11.5 transient transgenic embryos were generated by Cyagen Biosciences (Guangzhou, China) and analysed as described (Ogilvy et al., 2007).

### PU.1-YFP embryo generation and analysis

Mice were housed with free access to food and water. All procedures were in compliance with United Kingdom Home Office regulations and approved by the Local Ethical Review Committee and the Home Office. Timed matings were set up overnight between wild-type (CBA×C57BL/6)/F1 male and female and homozygous PU.1-YFP male and female mice (Kirstetter et al., 2006). Dissection and flow cytometry analysis were undertaken as described previously (Swiers et al., 2013).

## Supplementary Material

Supplementary Material
