# Supplementary material for: Single-cell analyses of regulatory network perturbations using enhancer-targeting TALEs suggest novel roles for PU.1 during haematopoietic specification
Source: Development. 2014 Oct;141(20):4018–30. doi: 10.1242/dev.115709 (PMC4197694; doi:10.1242/dev.115709)
Supplement: Supplementary Material [file supp_141_20_4018__index.html]

Supplementary Material 

# Single-cell analyses of regulatory network perturbations using enhancer-targeting TALEs suggest novel roles for *PU.1* during haematopoietic specification

## DEV115709 Supplementary Material

**Files in this Data Supplement:**

- **Supplementary Material**
