# Supplementary material for: Single-cell analyses of regulatory network perturbations using enhancer-targeting TALEs suggest novel roles for PU.1 during haematopoietic specification
Source: Development. 2014 Oct;141(20):4018–30. doi: 10.1242/dev.115709 (PMC4197694; doi:10.1242/dev.115709)
Supplement: Supplementary Material [file supp_dev.115709_DEV115709.pdf]

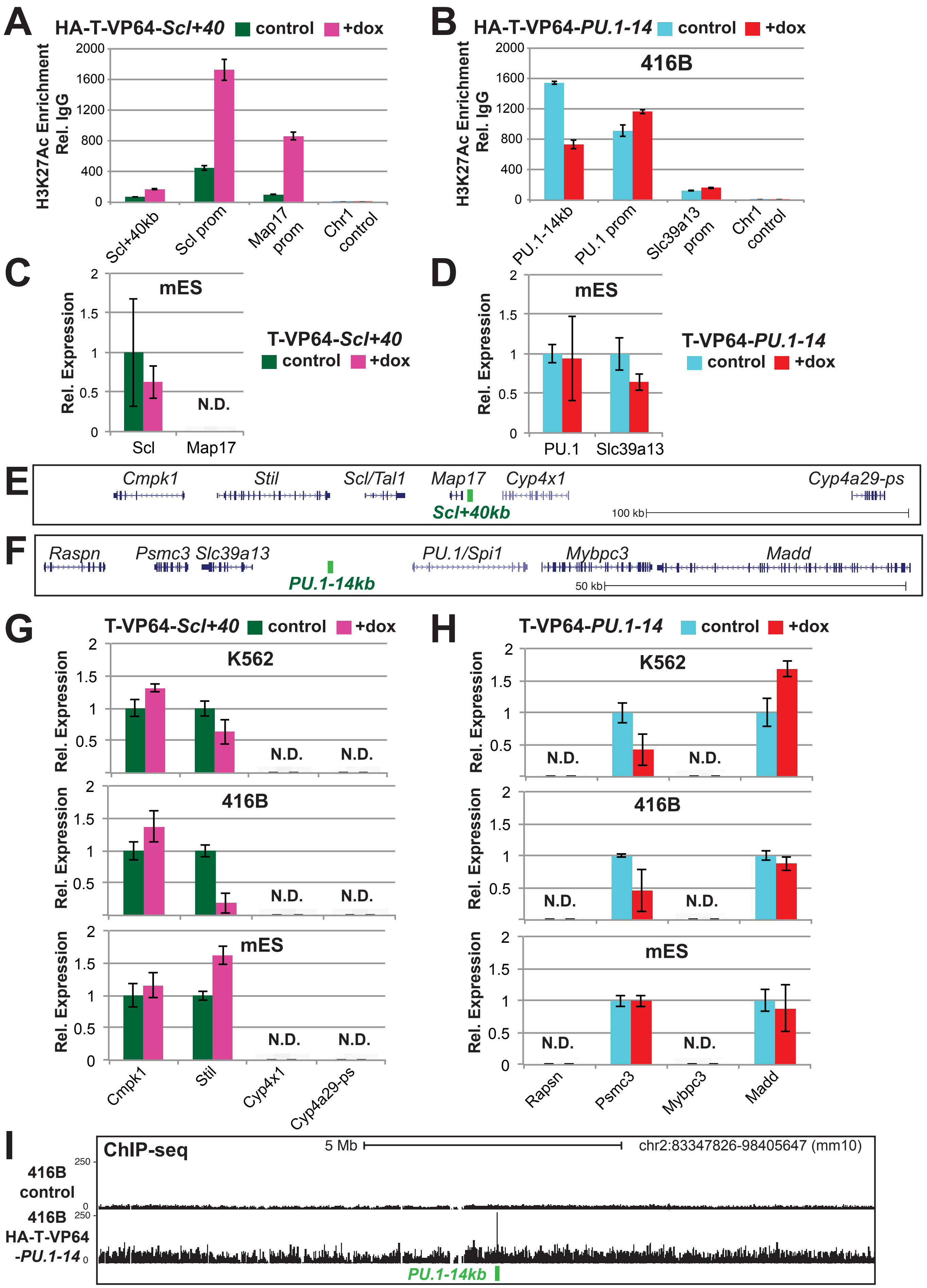

A
HA-T-VP64-Scl+40 control +dox
H3K27Ac Enrichment Rel. IgG
Scl+40kb
Scl prom
Map17 prom
Chr1 control
B
HA-T-VP64-PU.1-14 control +dox
416B
PU.1-14kb
PU.1 prom
Slc39a13 prom
Chr1 control
C
mES
Rel. Expression
N.D.
Scl
Map17
T-VP64-Scl+40 control +dox
D
mES
PU.1
Slc39a13
T-VP64-PU.1-14 control +dox
E
Cmpk1
Stil
Scl/Tal1
Map17
Cyp4x1
Cyp4a29-ps
Scl+40kb
100 kb
F
Raspn
Psmc3
Slc39a13
PU.1-14kb
PU.1/Spi1
Mybpc3
Madd
50 kb
G
T-VP64-Scl+40 control +dox
K562
416B
mES
Cmpk1
Stil
Cyp4x1
Cyp4a29-ps
H
T-VP64-PU.1-14 control +dox
Rapsn
Psmc3
Mybpc3
Madd
I
ChIP-seq
5 Mb
chr2:83347826-98405647 (mm10)
416B control
416B HA-T-VP64 -PU.1-14
PU.1-14kb

Figure S1, related to Figure 1:

(A) Effect of expressing HA-TALE-VP64 targeting the *Scl+40kb* (HA-T-VP64-*Scl+40*) for 48 hr in mouse 416B on histone 3 lysine 27 acetylation (H3K27Ac; relative to IgG enrichment) at the *Scl+40kb*, *Scl* promoter (prom), *Map17* prom and a control region on chromosome 1 (*chr1* control). HA-T-VP64-*Scl+40* expressed for 48 hr by addition of doxycycline (+dox; pink bars). Untransfected 416B used as control (green bars). Error bars are standard deviation of technical triplicates from one biological experiment.

(B) Effect of expressing HA-TALE-VP64 targeting the *PU.1-14kb* (HA-T-VP64-*PU.1-14*) in mouse 416B on histone 3 lysine 27 acetylation (H3K27Ac) ChIP-qPCR enrichment (relative to IgG enrichment) at the *PU.1-14kb*, *PU.1* prom, *Slc39a13* prom and *Chr1* control region. HA-T-VP64-*PU.1-14* expressed for 48 hr by addition of dox (+dox; red bars). Untransfected 416B used as control (blue bars). Error bars are standard deviation of technical triplicates from one biological experiment.

(C) Effect of expressing TALE-VP64 targeting the *Scl+40kb* (T-VP64-*Scl+40*) in mouse Ainv18 ES cells on *Scl* and *Map17* gene expression, normalised to *ActB*. T-VP64-*Scl+40* expressed for 48 hr by addition of dox and gene expression in +dox cells (green bars) determined relative to –dox control cells (pink bars). Error bars are standard deviation of technical triplicates from one biological experiment.

(D) Effect of expressing TALE-VP64 targeting the *PU.1-14kb* (T-VP64-*PU.1-14*) in mouse Ainv18 ES cells on *PU.1* and *Slc39a13* gene expression, normalised to *ActB*. T-VP64-*PU.1-14* expressed for 48 hr by addition of dox and gene expression in +dox cells (blue bars) determined relative to –dox control cells (red bars). Error bars are standard deviation of technical triplicates from one biological experiment.

(E) UCSC Genome Browser screen shot of RefSeq annotated genes within a ~300kb genomic window surrounding the *Scl+40kb* enhancer (highlighted in green).

(F) UCSC Genome Browser screen shot of RefSeq annotated genes within a ~150kb genomic winder surrounding the *PU.1-14kb* enhancer (highlighted in green).

(G) Effect of expressing T-VP64-*Scl+40* in human K562 (top), mouse 416B and mouse Ainv18 ES cells on *Cmpk1*, *Stil*, *Cyp4x1* and *Cyp4a29-ps* gene expression, normalised to *ACTB/ActB*. T-VP64-*Scl+40* expressed for 48 hr by addition of dox and gene expression in +dox/mCherry^{+} cells (green bars) determined relative to –dox/mCherry^{-} control cells (pink bars). Error bars are standard deviation of technical triplicates from one biological experiment. Not detected; N.D

(H) Effect of expressing T-VP64-*PU.1-14* in human K562 (top), mouse 416B and mouse Ainv18 ES cells on *Rapsn*, *Psmc3*, *Mybpc3* and *Madd* gene expression, normalised to *ACTB/ActB*. T-VP64-*PU.1-14* expressed for 48 hr by addition of dox and gene expression in +dox/mCherry^{+} cells (blue bars) determined relative to –dox/mCherry^{-} control cells (red bars). Error bars are standard deviation of technical triplicates. Not detected; N.D

(I) UCSC Genome Browser screenshot of HA antibody ChIP-Seq enrichment in a ~15Mb window surrounding the *PU.1-14kb* element in wild type (above) and T-VP64-*PU.1-14* expressing (below) 416B cells.

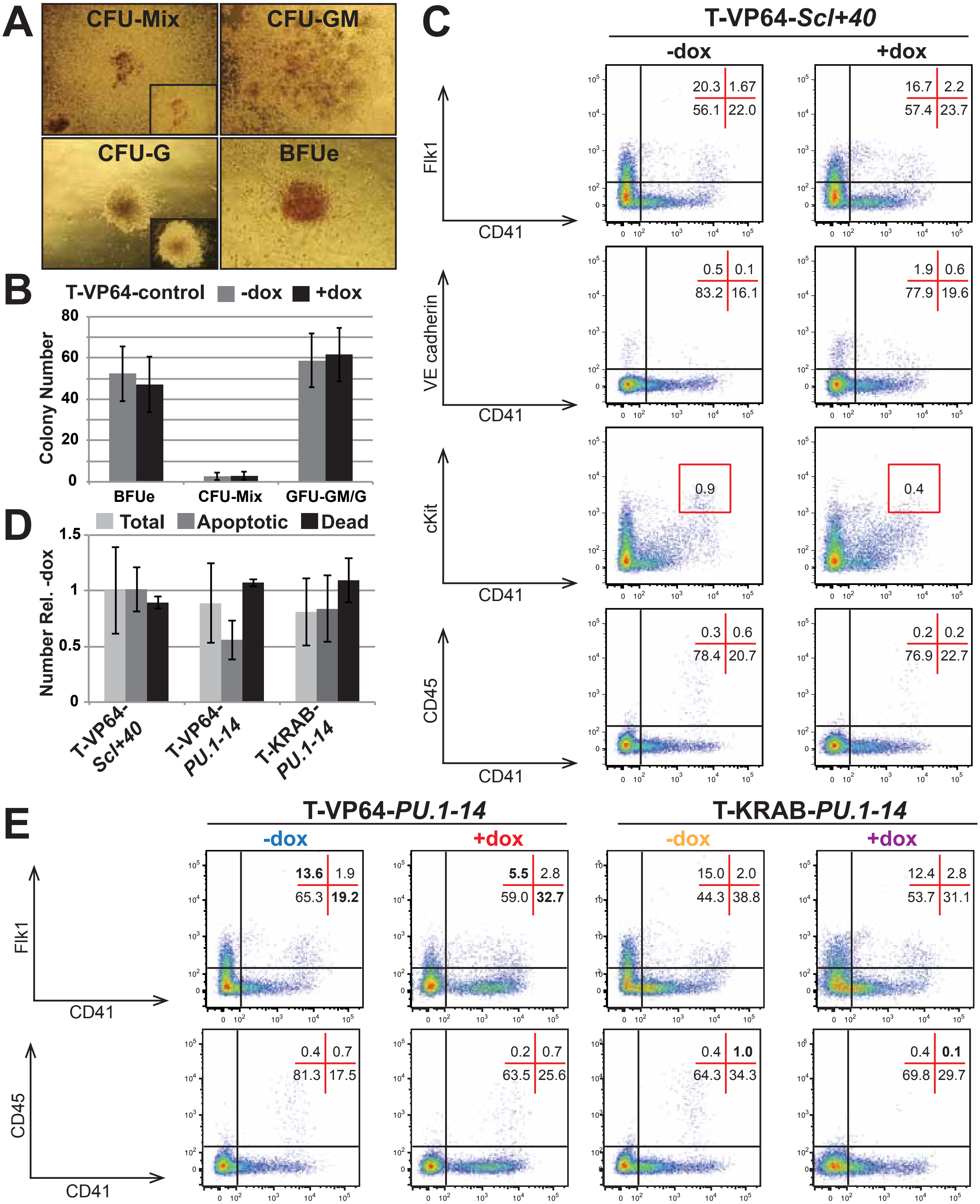

F

Population	T-VP64-PU.1-14 EB absolute cell numbers		
	(-)dox	(+)dox	p value
Flk1+	164633±80581	47503±41865	<0.01
CD41+	157083±135844	143550±115053	N.S
CD41+VEcad+	2213±2299	12852±11881	<0.05
CD41+cKit+	11445±8974	1945±1503	<0.05

G

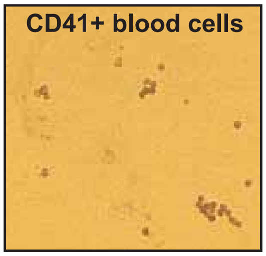

Figure S2

Figure S2, related to Figure 2:

(A) Representative images of haematopoietic colonies scored in methylcellulose CFU assays in Figures 2C and S2B. Red coloured erythroid colonies of at least ~30 small cells dispersed within small clusters with tight cell-cell junctions were scored as burst forming unit erythroid (BFUe). Colonies of at least ~50 small round bright cells (often tightly packed with grey centre) were scored as colony forming unit-granulocyte (CFU-G). Large colonies of over ~200 cells containing both granulocytes (as described above) and macrophages (large round cells, less bright than granulocytes and often more dispersed) were scored as colony forming unit-granulocyte macrophage (CFU-GM). Large colonies of over ~200 cells, densely packed, including red erythroid cells (similar to those described above) as well as at least two other lineages (usually granulocytes and macrophages described above) or megakaryocytes were scored as colony forming unit-mix (CFU-Mix).

(B) Representative haematopoietic colonies numbers from 1 x 10^5 day 6 EB cells derived from mouse ES cells inducible expressing a non-functional TALE-VP64 (due to mutations within the DNA binding domain), previously generated (Gao et al., 2013). As in Figure 2C, dox added to EBs day 4 to induce TALE-VP64 expression. Colonies grown in methylcellulose supplemented with SCF, IL-3, IL-6 and Epo. Error bars are standard deviation of technical triplicates. No statistically significant changes in CFU numbers were seen from three biological triplicates, as determined by the student t test.

(C) Flow cytometry plots of day 6 EB cells showing (from top to bottom) Flk1 vs. CD41, VE cadherin (VEcad) vs. CD41, cKit vs. CD41, CD45 vs. CD41). Representative staining patterns for a T-VP64-*Scl+40* expressing mouse ES cell line, both uninduced (-dox) and induced (from day 4; +dox). Distribution of cells within quadrant/gates shown as percentages.

(D) Total number (light grey bars), frequency of apoptotic (Annexin V^+ $DAPI^-$; dark grey bars) and frequency of dead (Annexin V^+ $DAPI^+$; black bars) T-VP64-*Scl+40*, T-VP64-*PU.1-14* and T-KRAB-*PU.1-14* expressing EB day 6 cells (+dox from day 4) relative to –dox controls. Error bars are standard deviation of three biological replicates. No statistically significant changes were seen from three biological triplicates, as determined by the student t test.

(E) Flow cytometry plots of day 6 EB cells showing Flk1 vs. CD41 (top) and CD41 vs. CD45 (bottom). Representative staining patterns for TALE-VP64 (left) and TALE-KRAB (right) targeting *PU.1-14kb* clones, both uninduced (-dox) and induced (from day 4; +dox). Distribution of cells within quadrant/gates shown as percentages.

(F) Table displaying absolute cells numbers for cells populations identified by flow cytometry in Figure 2D ± standard deviation from three biological replicates, and p values (using the student t test). N.S; not significant.

(G) Representative image of day 4 EB $Flk1^+$-derived colony containing haematopoietic (round, budding) $CD41^+$ (stained black) cells scored in Figure 2E. Colonies containing endothelial cells that stained weakly $CD41^+$ were not scored unless haematopoietic cells were also present.

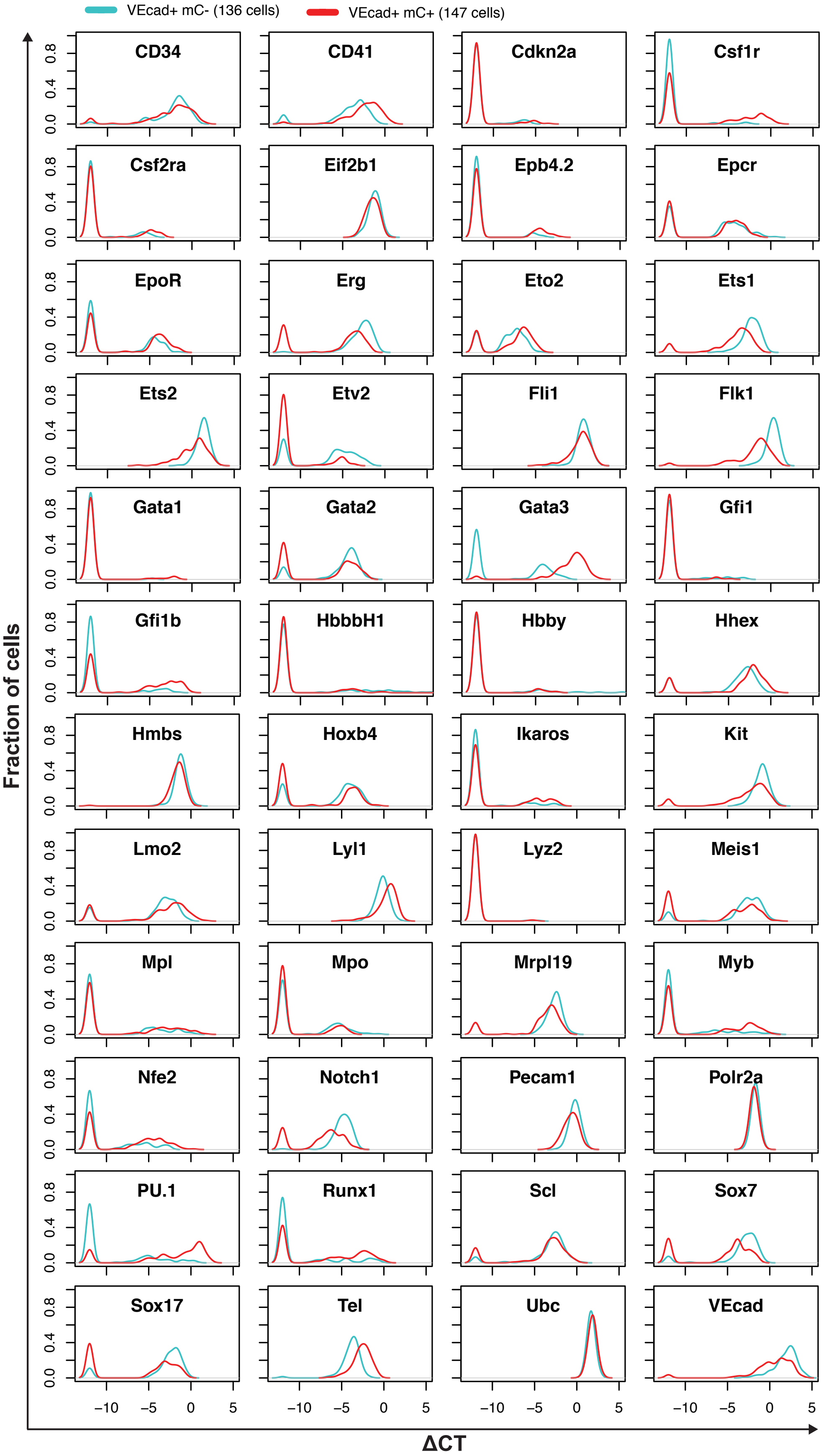

Figure S3

Figure S3, related to Figure 3:
Density plots of gene expression in day 6 EB VEcad[+] mCherry[-] (136 WT Ainv18; in cyan) and VEcad[+] mCherry[+] (147 Ainv18 expressing T-VP64-*PU.1-14*; in red) for all 48 genes analysed. The density indicates the fraction of cells at each expression level, relative to housekeeping genes (*Polr2a* and *Ubc*). Cells with non-detected gene expression set to -12.

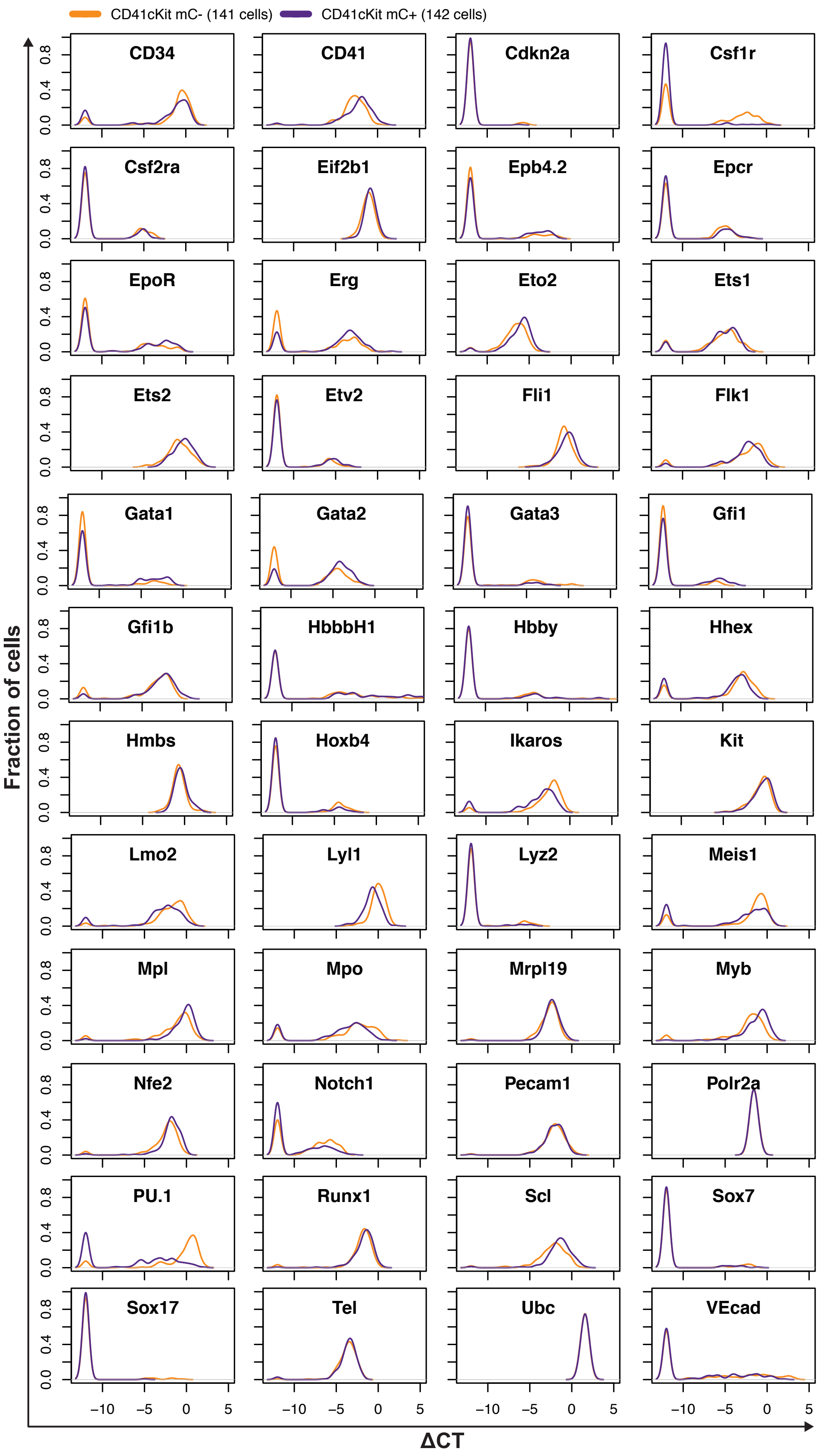

Figure S4

Figure S4, related to Figure 4:
Density plots of gene expression in day 6 EB CD41⁺cKit[hi] mCherry⁻ (141 WT Ainv18; in orange) and CD41⁺cKit[hi] mCherry⁺ (142 Ainv18 expressing T-KRAB-*PU.1-14*; in purple) for all 48 genes analysed. The density indicates the fraction of cells at each expression level, relative to housekeeping genes (*Polr2a* and *Ubc*). Cells with non-detected gene expression set to -12.

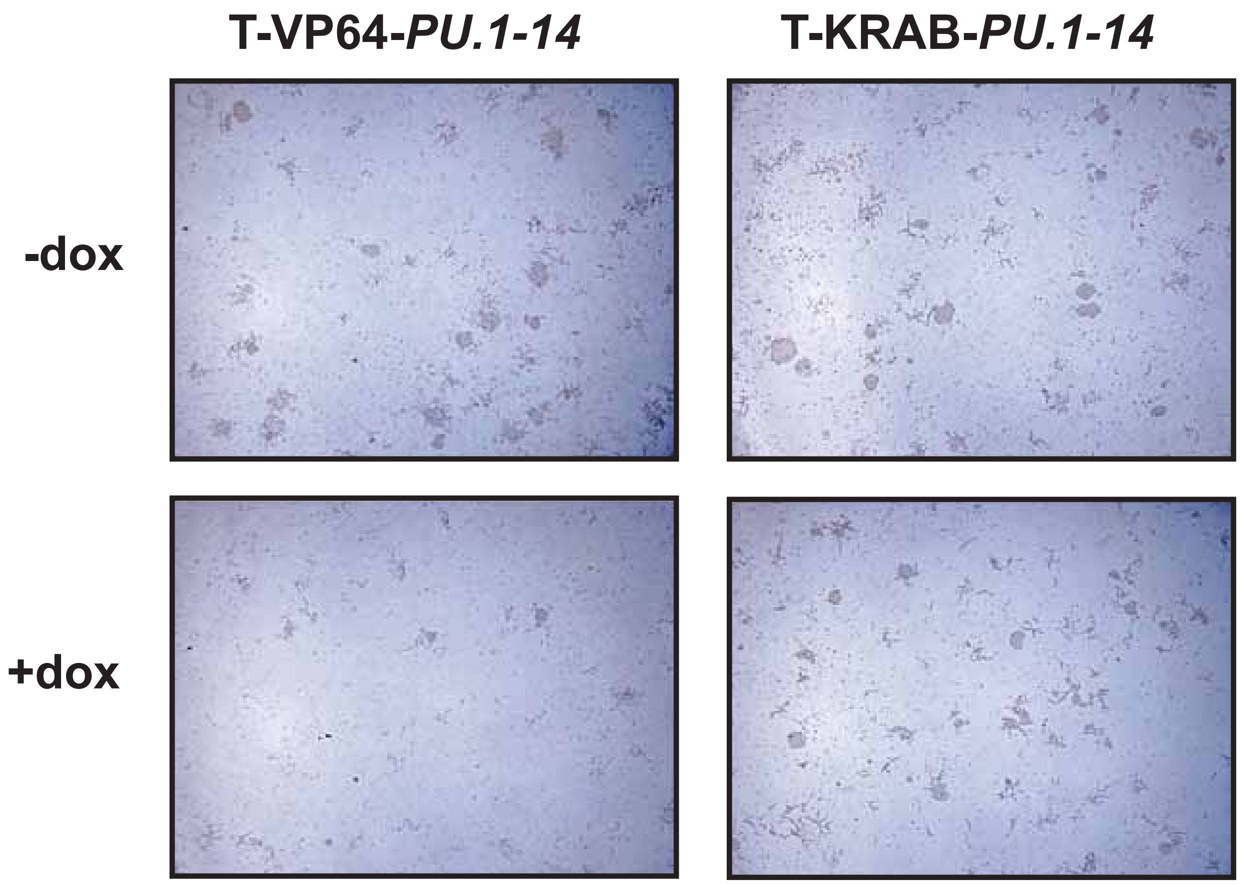

Figure S5, related to Figure 4:
Representative images of endothelial colony assays from day 6 EB VEcad[+] cells from T-VP64-*PU.1-14* or T-KRAB-*PU.1-14* ES cell lines as described in Figure 4.

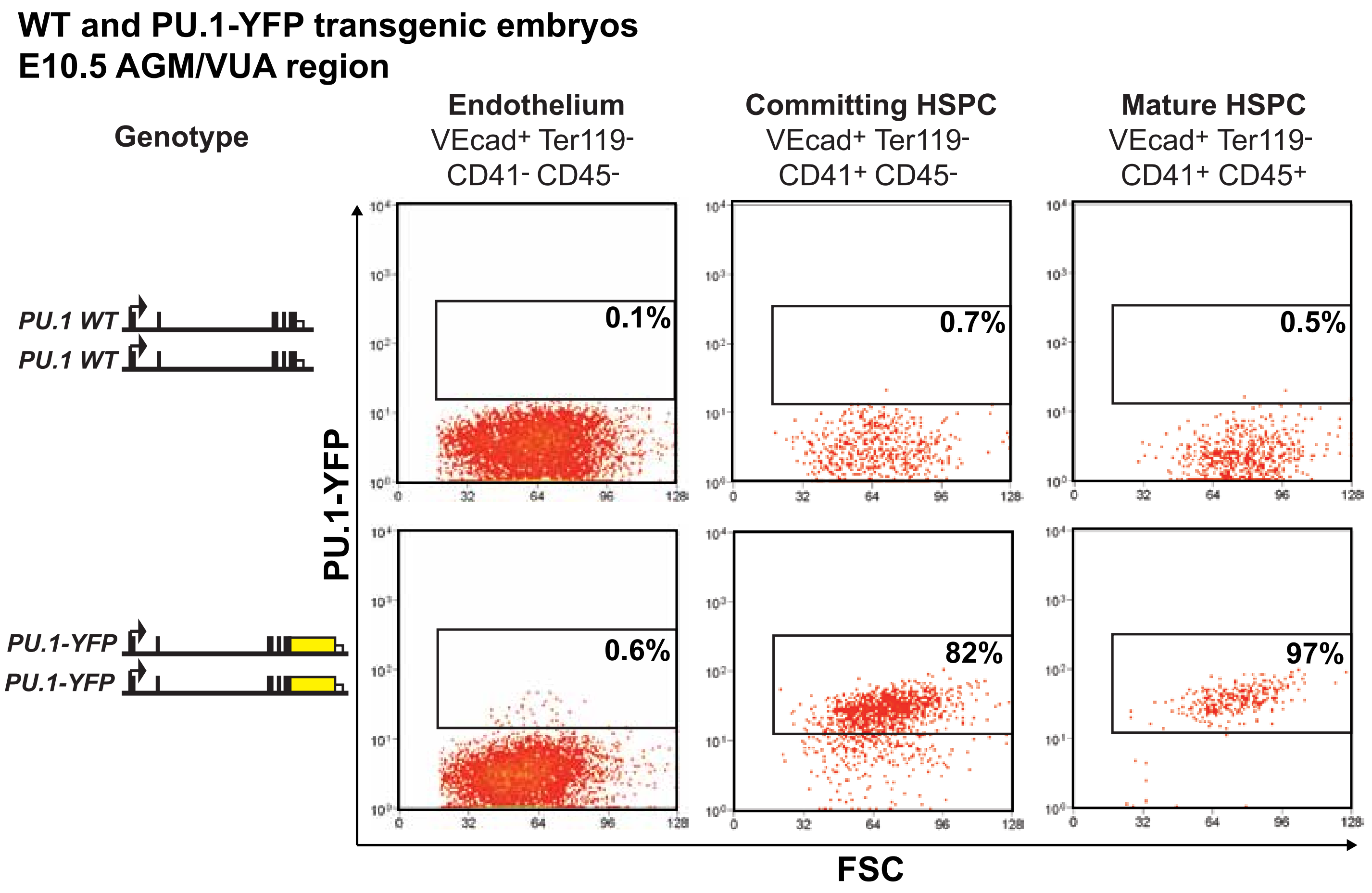

Figure S6, related to Figure 7:
Alternative analysis of flow cytometry data from Figure 6B,C, plots displaying PU.1-YFP expression vs. FSC for VEcad+ Ter119- CD41- CD45- (left), VEcad+ Ter119- CD41+ CD45- (middle) and VEcad+ Ter119- CD41+ CD45+ (right) populations from the AGM+VUA region of pooled E10.5 WT (above) and PU.1-YFP (below) embryos.

Supplementary Table 1: Partial Correlation Analysis

Download Table S1

Supplementary Table 2: Partial Correlation Analysis

Download Table S2

Supplementary Table 3: Flow Cytometry Antibodies

Antibody	Supplier	Cat. Number	Clone ID	Reactivity
CD16/32 (Fc block)	BD	553142	2.4G2	Mouse
CD41-PE-Cy7	Biolegend	25-0411-82	eBioMWReg30	Mouse
CD41-APC	eBioscience	17-0411	MWReg30	Mouse
CD45-APC	Biolegend	103112	30-F11	Mouse
Flk1-APC	BD	560070	Avas 12a1	Mouse
Flk1-PE	BD	561052	Avas 12a1	Mouse
VEcad-PE-Cy7	Biolegend	92055	VECD1	Mouse
cKit-APC	BD	553356	2B8	Mouse

Supplementary Table 4: RT-qPCR primers

**Primer name**	**Sequence**
Human ACTB forward	AGAGCTACGAGCTGCCTGAC
Human ACTB reverse	AGCACTGTGTTGGCGTACAG
Human SCL/TAL1 forward	TTCCCTATGTTCACCACCAA
Human SCL/TAL1 reverse	AAGATACGCCGCACAACTTT
Human MAP17 forward	TGCCTATGAGAATGTGCCG
Human MAP17 reverse	TGGACATCCATCCCATGTGC
Human PU.1/SPI-1 forward	CGGCTGGATGTTACAGGCGTG
Human PU.1/SPI-1 reverse	TCGTGCGTTTGGCGTTGG
Human SLC39A13 forward	TTCCCCTAGAGATGGGGACC
Human SLC39A13 reverse	GGCAGCAGATGCAGAAACAC
Human PSMC3 forward	ACAGACGTACTTCCTTCC
Human PSMC3 reverse	CCAATGAACATCTGCACCAG
Human RAPSN forward	GTACGACTCCGCCATGAGCA
Human RAPSN reverse	ATGGCATCCAGAGCCTTGTCC
Human MYBPC3 forward	GCTCTTCCAGACCCATCTCG
Human MYBPC3 reverse	CAGCGGGATGACAGGAAACA
Human MADD forward	TAGTGATCGTAGGGGCCAGG
Human MADD reverse	GCAGGGGAAACTCAGTGTGA
Human STIL forward	ATGCACATAACGTGGATCACG
Human STIL reverse	TCCATGCTCAAATCCACACC
Human CMPK1 forward	TCTCATGAAGCCGCTGGT
Human CMPK1 reverse	TCCTGCAGAAAGGTGTGTGT
Human CYP4X1 forward	TCAGGACACAAGCGTGGAGGTCTA
Human CYP4X1 reverse	TGCATAAGGATCATGGGTGCTGTT
Human CYP4A29-PS forward	CTGCTTTTCAAGGCAGCACA
Human CYP4A29-PS forward	CTGCAAGCAATGCCCAAAGA
Mouse Actb forward	TCCTGGCCTCACTGTCCAC
Mouse Actb reverse	GTCCGCCTAGAAGCACTTGC
Mouse Scl/Tal1 forward	CATGTTCACCAACAACAACCG
Mouse Scl/Tal1 reverse	GGTGTGAGGACCATCAGAAATCTC
Mouse Map17 forward	GTCCTTGTTGCAATCGTCTTC
Mouse Map17 reverse	GAGGAGTATCTGCCATCCATTC
Mouse PU.1/Sfpi-1 forward	AGAGCATACCAACGTCCAATG C
Mouse PU.1/Sfpi-1 reverse	GTGCGGAGAAATCCCAGTAGTG
Mouse Slc39a13 forward	TTGCTGGTCATTCCCCTGGA
Mouse Slc39a13 reverse	GTCCACCTAAGGCAAAGCTGA
Mouse Psmc3 forward	GACCGTGTGGGATGAAGCTG
Mouse Psmc3 reverse	CGCTGGACAATCTCTTCCGTG
Mouse Rapsn forward	ATATCGGGCCATGAGCCAGT
Mouse Rapsn reverse	TCACAACACTCCATGGCACTGC
Mouse Mybpc3 forward	TGAAGGGTCAGTCTCGGTAACC
Mouse Mybpc3 reverse	TCCTGTGGTCGCATCAGAAA
Mouse Madd forward	AAGAAACTGGGCATCCCTCG
Mouse Madd reverse	GAAGGGCACTGGACTTCTCC
Mouse Stil forward	GGTGATGATCAAGAGCCCGA
Mouse Stil reverse	ACCAGGTTCTTTGCTCTGCT
Mouse Cmpk1 forward	TCAGAAGCGCGTTGTATGCT
Mouse Cmpk1 reverse	AAAACGAACACGACCAACGG
Mouse Cyp4x1 forward	CCTGGACATAATAATGAAATGTGCTT
Mouse Cyp4x1 reverse	CTTCACGTAAGACTCATAGGTGCC
Mouse Cyp4a29-ps forward	CAGTGCACCATCTGGACCTC
Mouse Cyp4a29-ps reverse	GATTACGTAATAGTGGTCCCTCAGG

Supplementary Table 5: Taqman assays used for single cell gene expression

Protein name	Assay ID
Csf1r/c-fms	Mm01266652_m1
CD34	Mm00519283_m1
CD41	Mm00439768_m1
Cdkn2a	Mm00494449_m1
Csf2ra	Mm00438331_g1
Eif2b1	Mm01199614_m1
Epb4.2	Mm00469107_m1
Epcr	Mm00440993_mH
EpoR	Mm00438760_m1
Erg	Mm01214246_m1
Eto2	Mm00486780_m1
Ets1	Mm01175819_m1
Ets2	Mm00468977_m1
Etv2	Mm00468389_m1
Fli1	Mm00484409_m1
Flk1	Mm01222421_m1
Gata1	Mm00484678_m1
Gata2	Mm00492300_m1
Gata3	Mm00484683_m1
Gfi1	Mm00515855_m1
Gfi1b	Mm00492318_m1
Hbb-bH1	Mm00756487_mH
Hbb-y	Mm00433936_g1
hHex	Mm00433954_m1
Hmbs	Mm01143545_m1
Hoxb4	Mm00657964_m1
Ikaros	Mm01187882_m1
Kit	Mm00445212_m1
Lmo2	Mm01281680_m1
Lyl1	Mm01247198_m1
Lyz2	Mm01612741_m1
Meis1	Mm00487659_m1
Mpl	Mm00440310_m1
Mpo	Mm00447886_m1
Mrpl19	Mm03048937_m1
Myb	Mm00501741_m1
Nfe2	Mm00801891_m1
Notch1	Mm00435249_m1
Pecam1	Mm01242584_m1
Polr2a	Mm00839493_m1
PU.1	Mm00488142_m1
Runx1	Mm01213405_m1
Scl/Tal1	Mm01187033_m1
Sox17	Mm04208182_m1
Sox7	Mm00776876_m1
Tel	Mm01261325_m1
Ubc	Mm01201237_m1
VE cadherin	Mm00486938_m1

Supplementary Table 6: ChIP-qPCR primers

**Primer name**	**Sequence**
Scl promoter F	CATGCGCACTCCAGCCTC
Scl promoter R	CACACACCGCCCAGAAGC
Map17 promoter F	ACAAGGTAGCACAGGACAGG
Map17 promoter R	TCCAAAACACCACCCCATCC
Scl+40kb F	CTCTTCCTTATCGCCAGCTC
Sc+40kb R	CAGCTGGTGCGTTATCAGTT
PU.1 promoter F	GGGCCATTGGCTTCCTTAGA
PU.1 promoter R	AATAGCTGTTCAGGCCCCAC
Slc39a13 promoter F	AGAGTAGGACGGAAGTGGGTA
Slc39a13 promoter R	CTGTTGATCCCGGTTCCCG
PU.1-14kb F	GCTGTTGGCGTTTTGCAAT
PU.1-14kb R	GGCCGGTGCCTGAGAAA
Chr1 control	CATAGATGAAGCTGCCACATAGGT
Chr1 control	GTGGGCAAGGACAAAGCATTA

Supplementary Table 7: Genomic PCR cloning primers

**Primer**	**Sequence**
PU.1prom_F	taaagatctCACCAGAGGGGACTGAGAAG
PU.1prom_R	taaaagcttGCTGAGCTCCAGGTTGGTC
PU.1-14kb_F	taaggatccGCTTGGGTGCTGGACTTAGA
PU.1-14kb_R	taagtcgacGACCTTTCCCTTGGGTGAGC
PU.1-18kb_F	taaggatccGGAGGGCTGTGTCAGTCAGA
PU.1-18kb_R	taagtcgacCTTCTTCCATCCCACAGAGC
